# Geodetic imaging of magma ascent through a bent and twisted dike during the Tajogaite eruption of 2021 (La Palma, Canary Islands)

**DOI:** 10.1038/s41598-023-50982-9

**Published:** 2024-01-02

**Authors:** Monika Przeor, Raffaele Castaldo, Luca D’Auria, Antonio Pepe, Susi Pepe, Takeshi Sagiya, Giuseppe Solaro, Pietro Tizzani, José Barrancos Martínez, Nemesio Pérez

**Affiliations:** 1 Instituto Volcanológico de Canarias (INVOLCAN), Granadilla de Abona, Tenerife, Canary Islands Spain; 2 Instituto Tecnológico y de Energías Renovables (ITER), Granadilla de Abona, Tenerife, Canary Islands Spain; 3 Istituto Per il Rilevamento Elettromagnetico dell’Ambiente (CNR-IREA), Naples, Italy; 4 Nagoya University, Nagoya, Japan

**Keywords:** Geophysics, Volcanology, Natural hazards

## Abstract

On Sept. 19th, 2021, the largest historical eruption on the island of La Palma began, which had a significant scientific, social, and economic impact. The 2021 Tajogaite eruption was characterised by short precursors, lasting only 8 days. The seismicity started on Sept. 11th with a westward and upward migration of hypocenters. Permanent GNSS stations started recording deformation on Sept. 12th on the island's western side, which reached more than 15 cm just before the eruption. After the eruption onset, the ground deformation increased, reaching a maximum on Sept. 22nd and showing a nearly steady deflation trend in the following months. To better understand the dynamics of the eruption, we exploited a joint dataset of GNSS and Sentinel-1 SBAS time series along both ascending and descending orbits. To obtain the geometry of the causative source of the ground deformation, we combined the result of a preliminary non-linear inversion and the precise location of the seismicity. The resulting geometry of the source is that of a twisted dike bending eastward. We performed inverse modelling to obtain the spatiotemporal kinematics of the opening function of the dike. The forward modelling has been realised using a 3D finite-element approach considering the island's topography. Our findings reveal a close correspondence between the magmatic intrusion and pre-eruptive seismicity. The ascent of the magma occurred along two branches, and the rheology of a previously identified ductile layer strongly affected the magma propagation process. Finally, we found evidence of an early shallow deformation, which we interpret as the effect of ascending hydrothermal fluids. Our findings highlight the need for advanced modelling to understand pre-eruptive processes in basaltic volcanoes.

## Introduction

**Figure 1:**
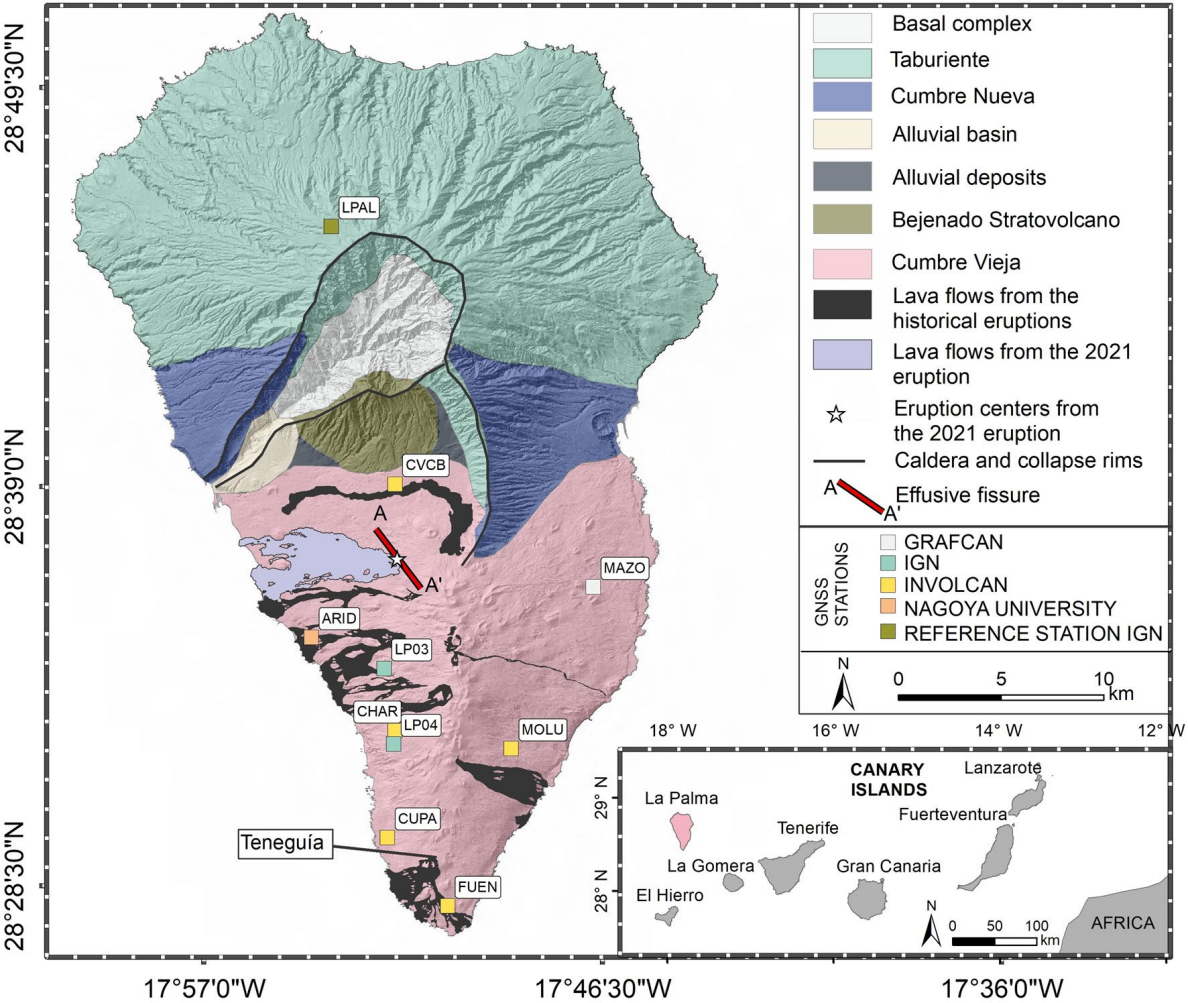
The geological map and the GNSS permanent stations location of La Palma. The lower right-hand side panel shows the location of the island of La Palma (in red) within the Canaries. The legend explains the meaning of the colour shades on the map and the GNSS station. The NW–SE alignment of the effusive fissure of the 2021 eruption is represented by the red line (A–A').

The Canary Islands are located off the northwest coast of Africa, 150 km from the African coastline (Fig. 1). The Canaries originated in the intraplate region of the African plate and extend along a 500 km wide alignment from East to West, in the framework of − 13° W and − 18° W longitudes and 27° N and 30° N latitudes. The formation of the Canaries started in the Oligocene and is still in process^1,2^. The most ancient islands are Fuerteventura and Lanzarote, the easternmost located of all Archipelago. Its formation continued with the direction to the West, being the most recent islands of El Hierro and La Palma.

La Palma Island formation started in the Pliocene, 4 My ago, as a seamount sequence that lasted about 1 My^2^. Between 3 and 2 My, the island emerged from the ocean, and a rapid elevation of the island caused the giant landslide. The subsequent formation of the Garafía Edifice and Taburiente Domain was also interrupted by a gravitational landslide. The formation of the Cumbre Nueva Domain was centred on the South of the Taburiente Domain.

Consequently, the Cumbre Vieja Domain started its formation 0.123 My ago^2^. This N–S volcanic ridge is still active and hosted all the historical eruptions of the island of La Palma. The latest eruption in La Palma in the past century (Teneguía eruption of 1971) emerged in the southernmost part of the island (Fig. 1).

On Sept. 19th, 2021, in the N.W. of the Cumbre Vieja ridge, a new volcanic eruption on the island started and lasted 85 days^3^. The eruption's consequences (gas emissions, a large volume of lava flows, and tephra dispersion) resulted in one fatality due to indirect causes and enormous economic and social losses^4^. The volcanic precursors, like ground deformation, seismicity, and gas emissions, were noticed 8 days before the eruption onset^3^. The permanent volcano monitoring stations of Instituto Volcanológico de Canarias (INVOLCAN) assessed the ground deformation and the pre-eruptive seismicity migration. The Global Navigation Satellite System (GNSS), the Sentinel-1 (S-1) satellite constellation, and seismic stations collected the data of the pre-and early-eruptive phases used in the present study. The first evidence of the magmatic intrusion began on Sept. 11th^3,5^, with a seismic swarm of volcano-tectonic character, with a depth of 10 km or less. The upward migration of the hypocenters lasted only 8 days until magma emerged to the surface. Considerable ground deformation appeared on continuous GNSS (cGNSS) stations of INVOLCAN on Sept. 12th and continued increasing to reach its maximum 3 days after the eruption onset.

This study analyses magmatic and hydrothermal sources that caused the pre-eruptive ground deformation in La Palma. To this aim, we processed a sequence of Synthetic Aperture Radar (SAR) images collected by the Sentinel-1 (A and B) satellites from January to November 2021. We used the multi-temporal differential interferometric SAR (DInSAR) and Small Baseline Subset (SBAS) method^6^. Subsequently, we analysed the InSAR-driven ground displacement measurements and the available cGNSS dataset from Sept. 8th to Sept. 28th, 2021. Accordingly, we performed a preliminary non-linear inversion to determine the dip of the shallow part of the dike. The geometry of the deeper part has been constrained by using the relocated hypocenters of D'Auria et al.^3^. Later on, following D'Auria et al.^7^ and Pepe et al.^8^, we applied the Geodetic Imaging technique to the DInSAR and cGNSS datasets to understand the ascent path of magma and the spatiotemporal dike aperture kinematics.

Previous studies evidenced the importance of advanced modelling of the ground deformation sources to understand the dynamics of a magmatic plumbing system^7–11^. The results of this study are supported by previous studies about local earthquake seismic tomography (LET) of La Palma^3^, Ambient Noise Tomography (ANT)^12^, deformation inverse modelling^10,11^, petrological studies^13^ and gravity surveys by Montesinos et al.^14^.

In this study, we denote the 8 days preceding the eruption as the pre-eruptive phase and the dates between Sept. 19th and 28th as the early-eruptive phase. Geodetic imaging proved to be an effective tool for understanding and visualising the complex magmatic ascent process on La Palma island during both phases.

## Results

### Preliminary non-linear inversion and dike geometry

Based on the results of previous studies^10^, we assume that the shallow part of the conduit consists of a southwestward dipping dike. Therefore, we performed a non-linear inverse modelling to constrain the dip of this shallow part of the conduit (see the Methods section for more details). Figures S1 and S2 in the supplementary materials represent the data, the model, and the residuals for each displacement map for both orbits.

**Figure 2:**
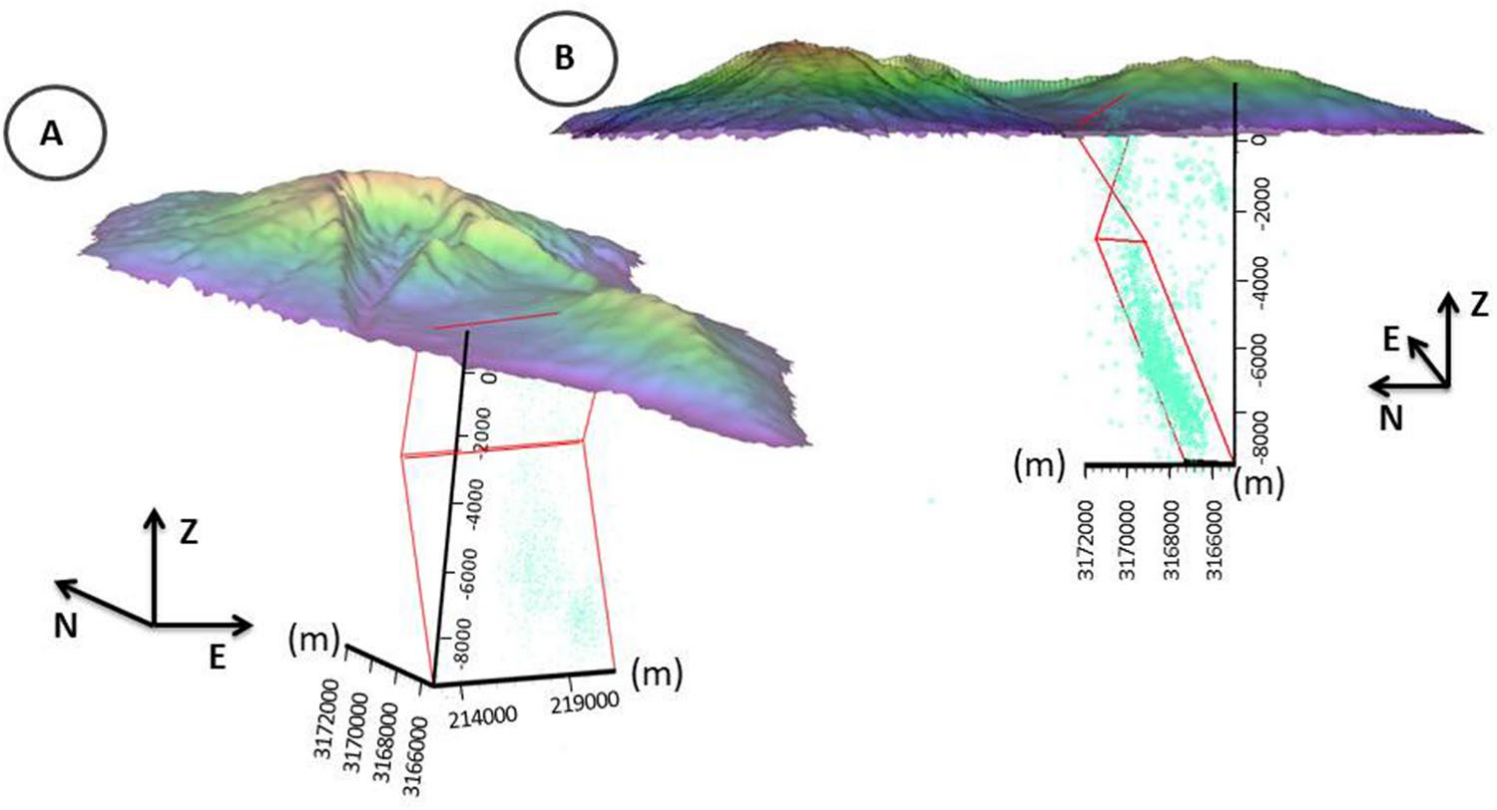
Tridimensional representation of the dike geometry and the topography of La Palma island. Panel A is a view from S.W., while B is from W. Earthquake hypocenters are represented as cyan circles. The axes are represented in meters (m).

We modelled the source using a simple rectangular dike geometry^15^, constraining the azimuth (125°), following the surface orientation of the eruptive fissures (Fig. 1)^16^. The retrieved best-fit value of the dip was 50° westward. The location and the geometry of the shallow dike have been adjusted to one of two shallow seismicity clusters corresponding to the dike ascent. However, the second shallow seismicity cluster is unrelated to the dike intrusion process. Previous studies^3,5^ demonstrated that it is related to hydrothermal activity triggered by the fluids released by the ascending magma. It is located to the South of the eruptive vents, and it is not relevant for modelling the dike geometry (Fig. 2).

However, this shallow dike alone cannot accurately describe the pre-eruptive intrusion process. The 3D pre-eruptive seismicity pattern^3^ shows a north-westward and upward migration of the hypocenters until Sept. 18th. This suggests that the lower part of the dike generated by the magmatic intrusion, starting at about 10 km depth, has an eastward dip. Therefore, a curved dike is a more appropriate geometry for the ground deformation source. To model the geometry of the lower part of the dike, we performed a geometrical fit with the hypocenters distribution using a simple rectangular geometry. The best-fit azimuth and dip are respectively 89° and 67° southward.

The final geometry results from merging these two dikes at a depth of about 3 km. This depth was selected based on the earthquake distribution, which shows a different trend starting from this depth^3^. The resulting geometry is that of a bent and twisted dike schematically shown in Fig. 2.

### Geodetic imaging

**Figure 3:**
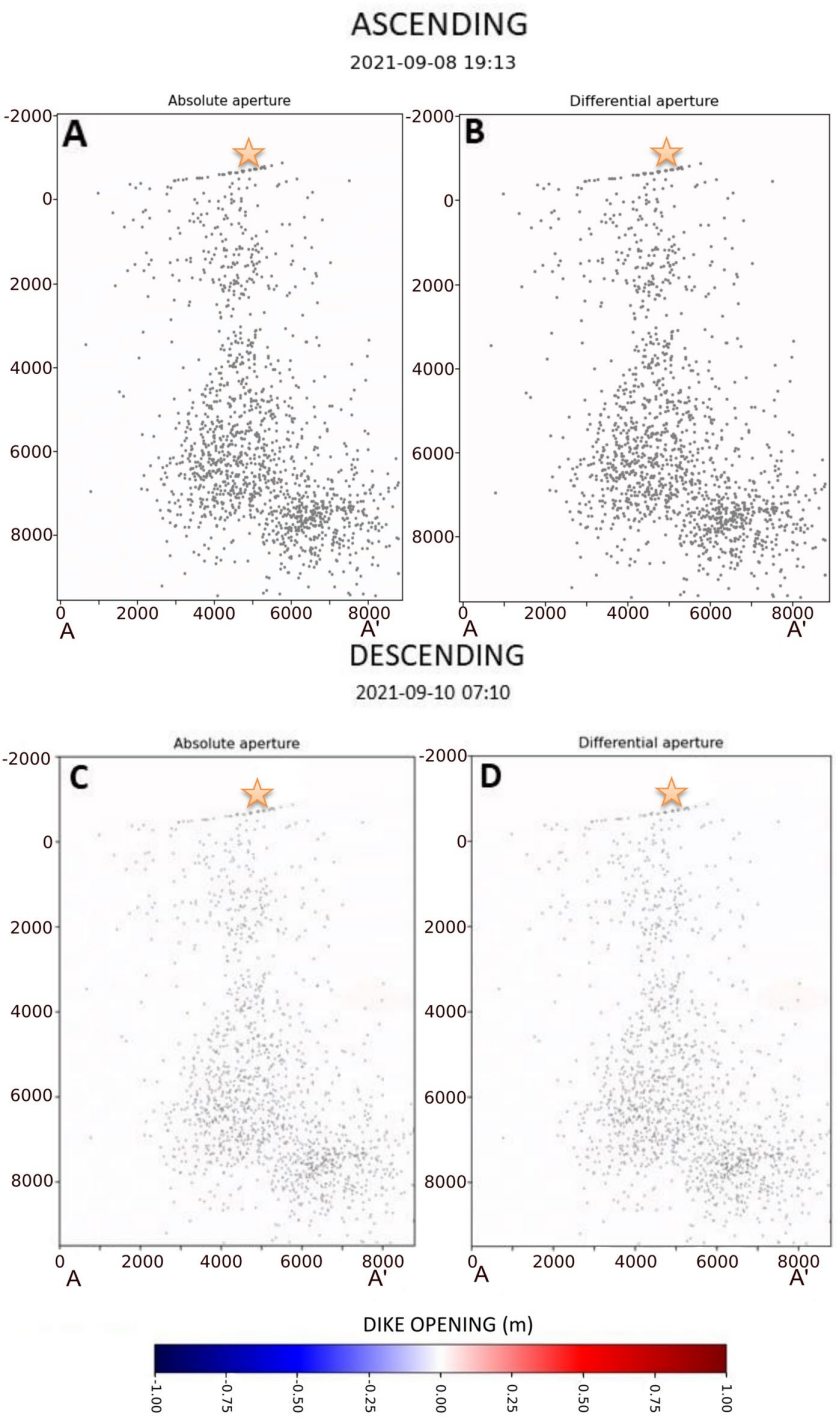
Distribution of absolute and differential dike opening for the 8th (ascending orbit) and Sept. 10th 2021 (descending orbit). Grey dots represent the projection on the dike of all the seismic events located between Sept. 11th and 28th. The seismic events colour in the images of Sept. 8th is represented with dark grey in order to highlight the total of earthquakes produced in the analysed period. Orange stars indicate the future location of the main eruptive vent.

**Figure 4:**
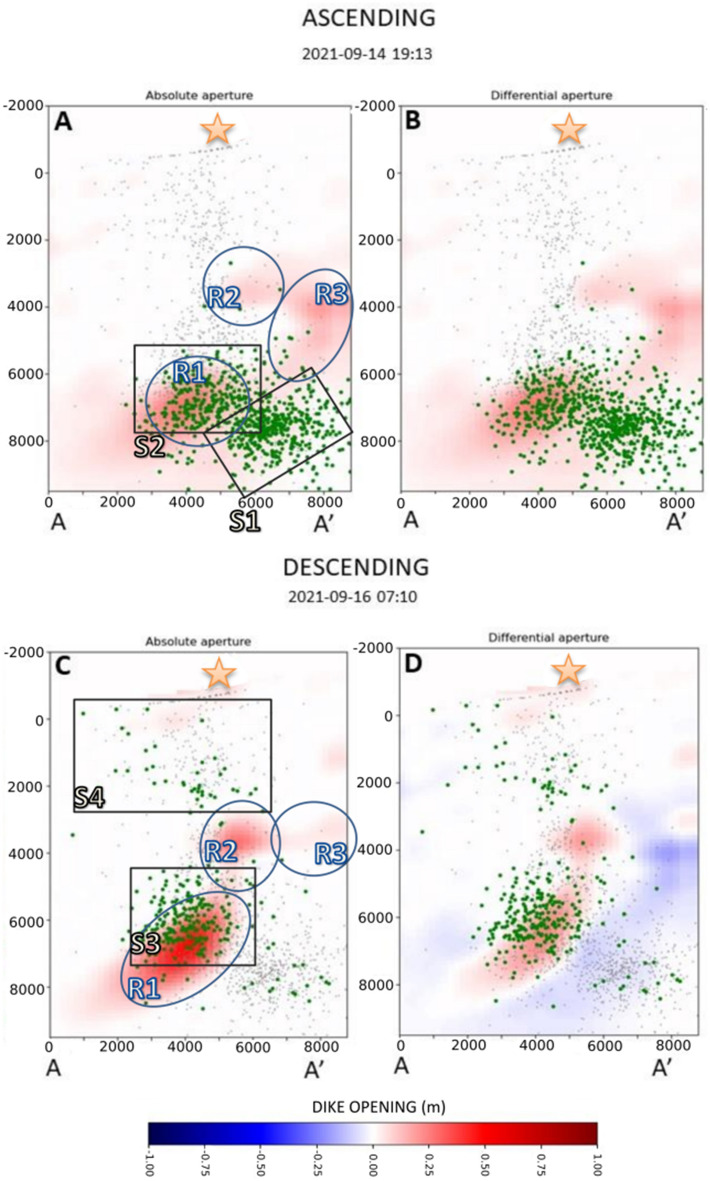
Distribution of absolute and differential dike opening for the 14th (ascending orbit) and Sept. 16th 2021 (descending orbit). Grey dots represent the projection on the dike of all the seismic events located between Sept. 11th and 28th, while green dots represent seismic events that occurred between two successive images. Blue ellipsoids and black squares represent, respectively, the features in the dike opening function and the clusters of seismic events discussed in the text. Orange stars indicate the future location of the main eruptive vent.

**Figure 5:**
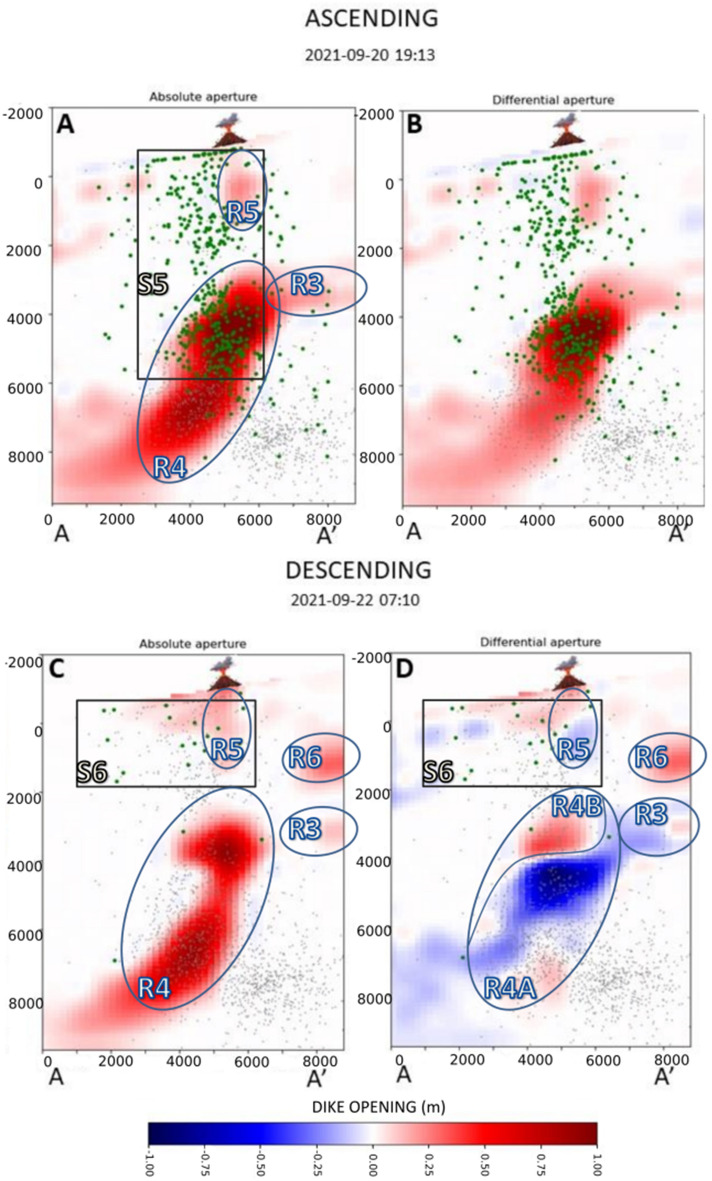
Distribution of absolute and differential dike opening for the 20th (ascending orbit) and Sept. 22nd, 2021 (descending orbit). The volcano symbol indicates the position of the main eruptive vent.

**Figure 6:**
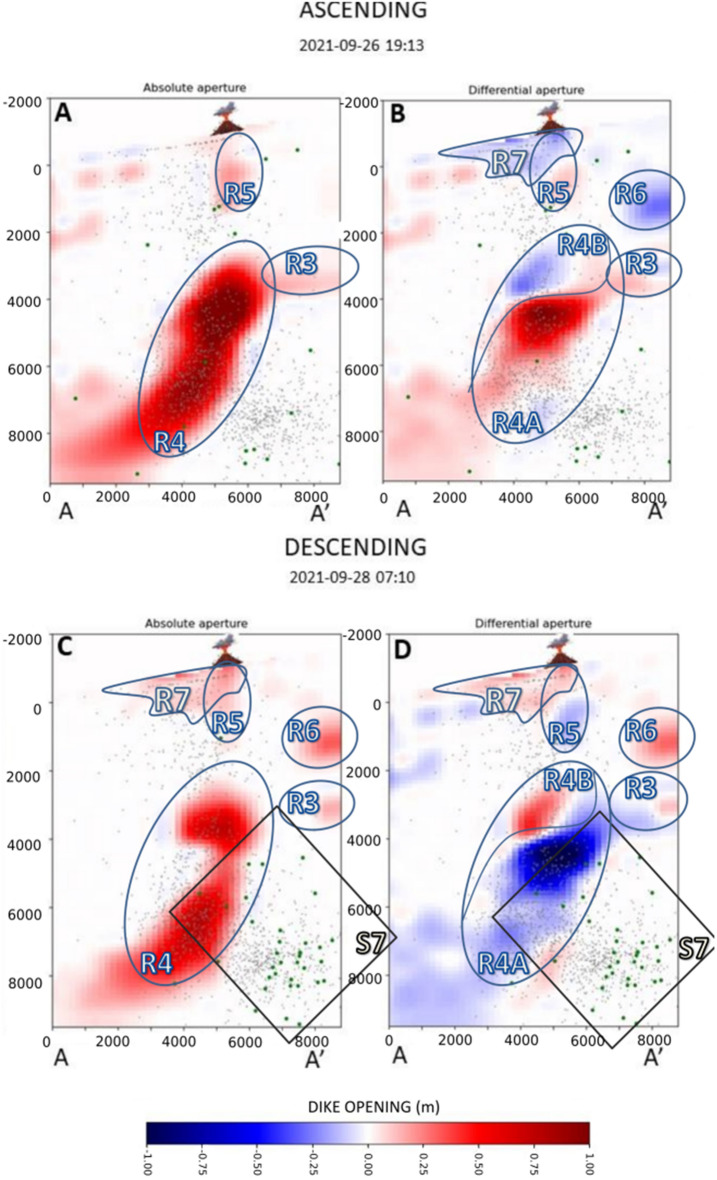
Distribution of absolute and differential dike opening for the 26th (ascending orbit) and Sept. 28th, 2021 (descending orbit). The meaning of the symbols is the same as in Fig. 5.

Using the dike geometry described in the previous section and applying the geodetic imaging technique described in "Geodetic imaging" section, we obtained a spatiotemporal imaging of the dike opening function. In the following, we describe in detail this result by showing both the absolute opening function (i.e., relative to the first image) and the differential one (i.e., relative to the previous image) (Figs. 3, 4, 5, and 6). For clarity, the dike opening function is shown on a 2D image. In each image, we also show the projection of all the earthquake hypocenters (represented with black dots) recorded between 11 and 28th Sept. 2021, while the earthquakes recorded between each image and the previous one are represented with green dots.

There is no visible deformation in the first two images of both orbits (Sept. 8th and 10th) (Fig. 3A–D). Until Sept. 11th, neither seismicity indicated relevant magma movement at depth.

Between Sept. 11th and 14th, the most relevant precursor of the approaching eruption was the north-westward and upward migration of the seismicity starting from a depth of about 10 km^3^. The ground deformation began to be significant on Sept. 14th, reflecting magma accumulation at a depth between 6 and 8 km (see R1 in Fig. 4A). The seismicity comprised two clusters located between 6 and 10 km. The first one, denoted S1, connects R1 with the magma chamber, located at more than 10 km depth by the local earthquake tomography^3^. The second cluster, S2, corresponds to the location of the magma accumulation zone in R1 (see Fig. 4A). The dike opening marked with the R2 is located along the primary magma pathway toward the surface. Conversely, the R3 is situated to the SW of the primary pathway, between 3 and 6 km depth.

The descending orbit from Sept. 16th shows that the dike opening R1 increased its magnitude and extends in depth between 5 and 8 km, with an approximate width of about 3 km (Fig. 4C,D). The R2 accumulation zone also increases its magnitude and area, reaching a diameter of approximately 2 km. The magma ascent was associated with a new cluster of seismicity migrating upward from S2 to S3. In this interval, we also observe a sparse, shallow cluster located between 0 and 3 km depth, represented by S4. The location of this cluster is spatially separated from the main seismicity related to the dike intrusion process^3,12^.

On Sept. 20th, 1 day after the beginning of the eruption, the previously observed accumulation zones R1 and R2 merged, forming a single accumulation zone extending between 3 and 9 km, with a width of about 3 km, indicated as R4 in Fig. 5. The seismic cluster S5 is shallower than the previous ones, extended from the surface until 6 km depth. In this image, we can also observe a shallow magma accumulation zone (R5 in Fig. 5), which extends between 0 and 2 km depth with a width of about 1 km and coincides with the location of the eruptive vent. We also observe an increase in the dike aperture of the accumulation zone R3 located at a depth between 3 and 4 km.

Since the beginning of the eruption, the strong volcanic tremor prevented the detection of low-magnitude seismicity^3^. However, on Sept. 22nd, the seismicity was mainly located at a shallow depth between 0 and 2 km, which is denoted as S6 in Fig. 5. At the same time, the accumulation zone R4 shows a marked change, with a decrease in the dike opening in the lower part and an increase in the upper part (see R4A and R4B in Fig. 5D). We also observe a decrease in the opening in the R3 zone. We also observe the appearance of a new accumulation zone (R6), between 2 and 4 km, located right above R3 Fig. 5C,D.

In the image of Sept. 26th, we observe a marked decrease in the dike opening right beneath the vent (R7 in Fig. 6). The zone R4 shows a reversal in its behaviour, with replenishment of its lower part. Analogously, the zones R3 and R6 show a similar reversal.

On Sept. 28th, we observed a renewed increase in the magnitude of R7, R6, and R3. The zone R4 shows a deflation, except for its upper part, and a significant reduction in width. On this date, we also observed the appearance of a deeper seismicity, located mainly below 6 km depth (Fig. 6C,D).

## Discussion

The complex dike geometry depicted in this work results from a joint analysis of geodetic and seismic data. A straightforward evidence that the geometry we depicted is realistic comes from the time series of horizontal cumulative displacement of the ARID station (see supplementary Fig. S14). It can be seen that until Sept. 18th, the displacement is mainly toward the W. After it changes abruptly to SW because the magma reached the upper part of the conduit, having a different orientation.

Different factors can affect the propagation of dikes: the stress field, the mechanical properties of the rocks, and the buoyancy of the magma^17^. First, we notice that the dike bends around a high-velocity body identified by the seismic tomography model of D'Auria et al.^3^. This can explain the north-westward migration of the intrusion during the pre-eruptive phase. The subsequent deviation toward the East and the development of its twisted geometry can be explained, taking into account the internal stress field of the volcano. Following Dahm et al.^18^ and Maccaferri et al.^19^, gravitational loads make magmatic intrusions move towards higher topography zones. In the case of La Palma, the highest altitudes are located along the N–S dorsal of the Cumbre Vieja domain (Fig. 1). Therefore, the eastward bending is compatible with the effect of gravitational loads due to the topography of the volcano. Actually, the majority of the historical and prehistoric vents of Cumbre Vieja are located close to the summit of the ridge^1^.

**Figure 7:**
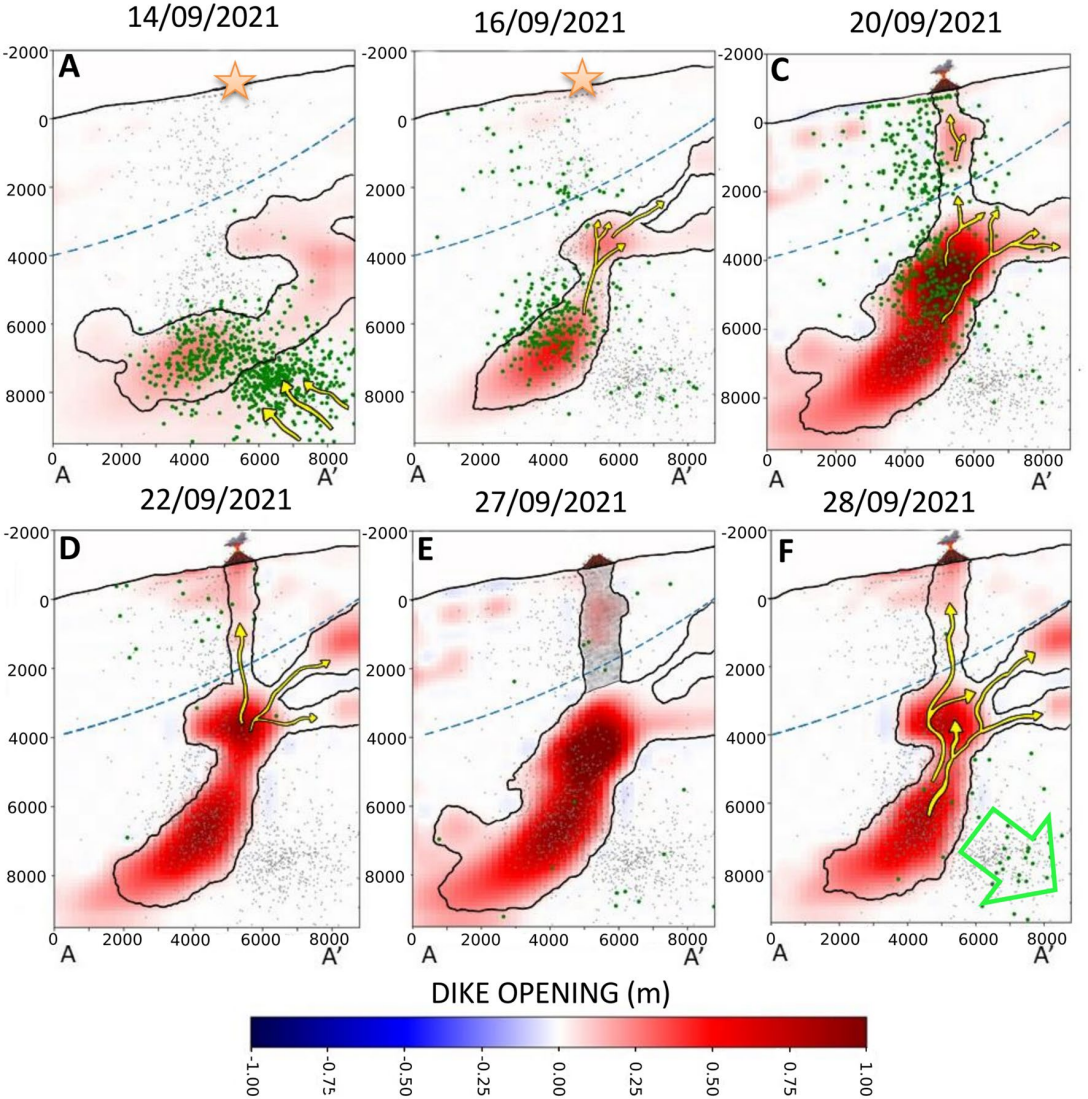
Schematic model of the plumbing system dynamics for key dates. Orange stars represent the location of the future site of the volcanic vent in the days preceding the eruption. The symbols of the volcano represent the location of the actual volcanic vent. Yellow arrows represent the magma ascent directions, while the green arrow indicates the incipient collapse of the magma reservoir. The blue dashed lines represent the limit of the rheological boundary discussed in the text.

The geodetic imaging results (Figs. 3, 4, 5, and 6) give a detailed overview of the kinematics of the magma movement within the dike and its relationship with seismicity. In Fig. 7, we represent, with a schematic cartoon, our interpretation of this process on some key dates. Our study reveals that the magma started accumulating beneath the Cumbre Vieja volcano at a depth of 6–8 km (zone R1 in Fig. 4) at least 5 days before the eruption (Sept. 14th). The simultaneous seismicity, occurring between 7 and 10 km (S1 in Fig. 4), possibly reflects the nucleation of the dike from the huge magma chamber, identified by D'Auria et al.^3^, beneath 10 km depth. On the other hand, the cluster S2 can be related to the local stress field perturbed by the accumulation of magma within the zone R1 (Fig. 7A). At the same time, the zones R2 and R3 seem to evidence a further minor accumulation zone beneath 4 km depth (Figs. 4A and 7A). Considering previous studies concerning the internal structure of La Palma^3,20,21^, we know that the first few km of the crust beneath Cumbre Vieja is characterised by low seismic velocities corresponding to low resistivity and low-density values. As discussed by Rivalta et al.^17^, the presence of crustal layering can significantly affect the dike propagation speed. We postulate that the different rheology of the first few km caused a temporary decrease in the dike ascent rate, causing the local accumulation in R2 and R3 (Figs. 4A and 7A).

The seismicity focused on two clusters on Sept. 16th (see Figs. 4C and 7B). The deeper one (S3) occurred between 4 and 7 km deep and is located atop the accumulation zone R1. This cluster possibly reflected the pressurisation of R1 and the upward propagation of the crack tip. At the same time, we observe an increase in the dike opening in R2 and R3, which we interpret as the transfer of magma toward a blind lateral branch of the main dike (Fig. 7B). Conversely, cluster S4 does not seem to be directly associated with a magmatic process. Following Cabrera-Pérez et al.^12^, we interpret this cluster as related to the ascent of the hydrothermal fluids exalted from the magma itself that generated the pressurisation of a shallow hydrothermal system. The presence of this hydrothermal system has already been highlighted by previous studies^3,21,22^. Furthermore, the study of Pankhurst et al. (2021) revealed that the first stages of the eruption presented more hydrated minerals, meaning that the ascending magma was fluid-rich.

The image of Sept. 20th is the first after the beginning of the eruption. The dike opening function clearly shows the opening of the pathway toward the eruptive vent (zone R5 in Figs. 5 and 7C). The seismicity pattern indicates that the magma approached the surface very quickly during the morning of Sept. 19th^3^. This rapid acceleration in the magma ascent rate when approaching the surface has been studied by Rivalta and Dahm^23^, which explained the physical mechanism of this process in terms of depth-dependent fracture toughness. The secondary blind branch R3 in the southeastern direction of the main dike was also increasing its aperture. As mentioned before, we believe this branch stopped its upward movement by a rheological boundary. Secondary branches departing from the main magmatic reservoir were also observed by Fernández et al.^11^. Their model shows two branches of magma that appeared due to zones of structural weakness in the crust. Montesinos et al.^14^, using gravity data, showed the possible appearance of a blind magma ascent path in the Jedey zone that could coincide with the R3 branch found in the present study. Also, the observed secondary branch R3 was possibly linked to the sill-like source mentioned by De Luca et al.^10^. Also, Muñoz et al.^16^ suggest that the dike developed multiple paths that could not reach the surface due to complex factors.

The main changes observed in the image of Sept. 22nd are the appearance of a further lateral branch (R6 in Figs. 5 and 7D) and a change in the magma distribution within the main feeding conduit R4 (Fig. 5D). We also observe the persistence of the lateral branch R3 (Figs. 5D and 7D).

Between Sept. 22nd and 26th, there was a visible reduction in the dike opening in the shallow part of the conduit (Figs. 6 and 7E). On Sept. 27th, a temporal stop of the eruptive activity was observed, associated with a marked drop in the volcanic tremor amplitude lasting about 10h^3,24,25^. The reduction observed in the image of Sept. 26th may be a precursor of the partial conduit collapse, which caused the temporary stoppage of volcanic activity the next day. Also, the shallower part of the main magmatic conduit (R4B) and secondary branch R6 shows a reduction possibly related to the lack of magma in the portion of the conduit located above 4 km depth.

The eruptive activity resumed in the afternoon of Sept. 27th. The image of Sept. 28th shows a dike aperture similar to Sept. 22nd, indicating that the primary process of the magma accumulation in the shallow crust went back to the initial scenario. However, as shown by Pankhurst et al.^13^, the magma erupted after Sept. 27th proceeded from a deeper reservoir, as testified by petrological analysis. Therefore, we believe that the renewed eruptive activity was driven by the arrival of a new magmatic batch with a more primitive composition. This also agrees with the appearance of a new seismic cluster (S7 in Fig. 6C,D), which has been interpreted by D'Auria et al.^3^ as the effect of the collapse of a magmatic reservoir located below 10 km depth because of its emptying due to the withdrawal of magma.

The modelling of the ground deformation source associated with the 2021 Cumbre Vieja eruption has already been analysed in previous studies, although using different approaches. In the following, we remark on the similarities and differences between their findings compared with the results of our study. De Luca et al.^10^ used a combination of elementary sources to perform a static imaging of the plumbing system. We used a complex geometry, using finite-element modelling, to image the spatio-temporal evolution of the plumbing system before and during the earliest phases of the eruption. De Luca et al.^10^ showed the existence of the sill-like source during the pre-eruptive phase and the presence of two dike-like sources active during the co-eruptive phases. The sill-like source was located at the 4675 m depth b.s.l. and was active between the 8th and 16th of September, corresponding mainly to the temporary accumulation of magma in its path towards the surface. This sill proposed by De Luca et al.^10^ can be well explained by one of the lateral blind branches resulting from our inversion. Secondly, in the co-eruptive phase, they found evidence that the shallow magmatic plumbing system feeding the eruption was composed of two dikes and sills interconnected to the main reservoir, as also evidenced by the present study with the interconnection of the dike to the lateral branches during the pre- and early-eruptive phases. Their models encompass the 10 km depth limit, as well as the model presented in this study. However, De Luca et al.^10^ only provided a static model of the ground deformation source, although the overall dike opening they retrieved matches pretty well with our image of Sept. 22nd (Fig. 5).

Conversely, Fernández et al.^11^ analysed the spatiotemporal evolution of the ground deformation source. However, they used a completely different modelling approach based on an improved version of the 3D multisource modelling algorithm of Camacho et al.^20^, which approximates the ground deformation sources as a combination of elementary pressure and fault slip sources. We believe this approach to be not entirely appropriate with volcanological observations of the Tajogaite eruption, which clearly evidenced a dike as the most likely geometry, at least for the shallow plumbing system. Therefore, in our approach, we tried to reproduce a physically realistic geometry and mechanism for the causative source of ground deformation. Also, their model evidences the deep source southward to the eruptive vent, as evidenced by the models presented in this study.

Additionally, we found the beginning of the deformation process related to the magmatic intrusion on Sept. 12th, while Fernández et al.^11^ found evidence of magma accumulation that started in May of 2021. However, their overall results are in agreement with our findings, especially concerning the presence of lateral branches in the plumbing system. Additionally, our approach allowed a direct computation of the dike opening function and established its temporal and spatial relationship with the seismicity.

Montesinos et al.^14^ used gravimetric and GNSS data acquired before and after the eruption and took into account the pre-eruptive seismicity to constrain the geometry of the plumbing system. They determined a complex geometry of the feeding system composed of interconnected dikes and sills. Their model also evidences the presence of a lateral blind branch of the plumbing system. Furthermore, they highlighted the temporary ascent of the magma on Sept. 14th due to the presence of horizontal layering within the crust.

To assess the reliability of our findings, we performed several checkerboards and tests over the synthetic dataset. In Figures S3, S4, S5, and S6 in the supplementary material, we represent the checkerboard test results for different spatial resolutions. We notice that our dataset is able to resolve anomalies of about 1 km size until a depth of 2 km (Fig. S3 in the supplementary material), anomalies of 2 km until about 4 km depth (Fig. S4 in the supplementary material), anomalies of 2.5 km until about 5 km depth (Fig. S5 in the supplementary material) and anomalies of 5 km along the whole model, until a depth of 10 km (Fig. S6 in the supplementary material). This confirms that our model is able to resolve the features described above. Furthermore, we conducted some additional synthetic tests to understand the limitations of our approach better. In Figure S7 of the supplementary material, we see that, except for the lower left corner, our inverse method is able to detect the presence of magma along the whole domain. However, in Figures S8 and S9 of the supplementary material, we observe a clear decrease in the spatial resolution at depth. This may justify the lack of evidence of the connection between the main magma conduit (R4) and the deeper magma chamber. Finally, in Figure S10 of the supplementary material, we perform a synthetic test over a realistic geometry of the magmatic system, showing that our inverse model is able to retrieve all the relevant features.

We also need to mention, that the difference in the acquisition geometry between ascending and descending orbits can slightly affect the results. Actually, some of the minor variations observed in the differential dike aperture models can be artefacts related to this effect.

## Conclusions

We propose a novel model of the spatiotemporal evolution of the magmatic system preceding and accompanying the first 10 days of the 2021 Cumbre Vieja eruption. For this purpose, we applied a Geodetic Imaging technique^7,26^ to reconstruct the kinematics of the plumbing system during the pre- and early-eruptive phases. The main finding of our study is that the causative source of the ground deformation was a dike with a bent and twisted geometry connecting a magmatic reservoir located below 10 km depth with the surface. Its azimuth changed from E-W in the deepest parts to NW–SE on the surface, while its dip changed from southward to southwestward.

We found an excellent agreement between the temporal evolution of the dike opening and the upward migration of pre-eruptive hypocenters. The upward propagation of the magma was very rapid (about 8 days) and strongly accelerated during the last day. The overall geometry of the dike intrusion process shows the presence of at least two blind lateral branches whose propagation stopped before reaching the surface. The eruption's onset is clearly evidenced by a dike opening right beneath the eruptive vent accompanied by intense shallow seismicity. On Sept. 27th, the eruption stopped for a few hours. We interpret it as an effect of a temporary collapse of the dike, as confirmed by the dike opening model, which shows an incipient collapse already starting the day before.

In conclusion, we state that the Geodetic imaging technique is an excellent tool for better understanding magma ascent processes. Our results provide evidence of the complexity of the dike propagation processes and the temporal changes in the shallow plumbing system before and during an eruption.

## Data and methods

### Data and processing of GNSS time series

**Figure 8:**
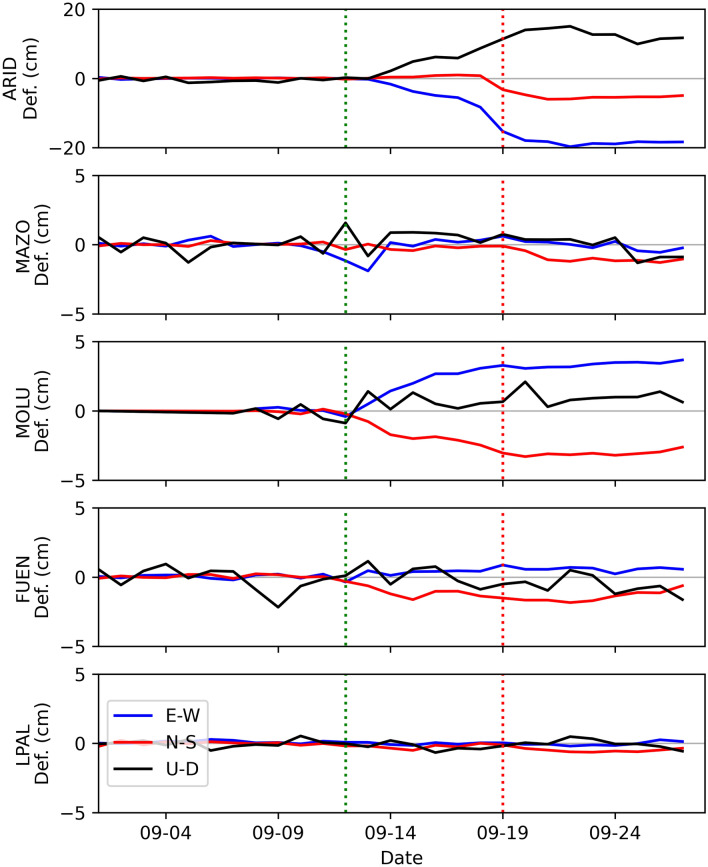
Solutions of some GNSS permanent stations solutions. Blue, red, and black horizontal lines show the E–W, N–S, and U–D components of deformation, respectively. The vertical red dotted line marks the day the eruption began, while the green dotted vertical line marks the day when the first significant deformation was recorded at stations MOLU and FUEN.

In this work, we used the permanent GNSS stations in La Palma island belonging to the Instituto Volcanológico de Canarias (INVOLCAN), the Nagoya University, and GRAFCAN (Fig. 1). Solutions are analysed by *GAMIT/GLOBK Software*^27^. For processing, we used a total of 27 stations. We removed the regional tectonic component from the solutions using the Nubian plate reference described by Saria et al.^28^. We also used solutions from three stations of Instituto Geográfico Nacional (IGN) denoted LP03, LP04, and LPAL. We selected these three stations of the IGN as the data from other stations of the IGN are not public. Figure 8 shows the time series of some of the stations used in this study.

In Figure S11 (in the supplementary materials), we compare data and the synthetic model resulting from the Geodetic Imaging technique for the three components of the GNSS data for all the stations used for this study. The stations closest to the eruptive vent (ARID) experienced the most significant deformation (Fig. 8). The deformation of ARID commenced on Sept. 14th (see Fig. 1) with 2.2 cm in the vertical component. It continued increasing in the following days, and on the day of the eruption, on Sept. 19th, the vertical deformation was already 11.4 cm, 15.3 cm toward the West, and 3.2 cm to the South. During the first week of eruption, the deformation continued increasing until it reached its maximum value in the ARID station, reaching 15.0 cm in the vertical component on Sept. 22nd (Fig. 8). Some GNSS stations at a higher distance from the eruptive vent also showed a deformation signal caused by the magmatic processes. Stations MOLU and FUEN (Fig. 1) began showing significant deformation on Sept. 12th. MOLU reached its maximum on Sept. 28th with 3.6 cm towards the East, 2.6 cm to the South, and 0.6 cm in the vertical component (Fig. 8). The deformed values did not return to their pre-eruptive stage and fluctuated during the eruption, showing a nearly steady deflation in the following months.

### DInSAR Sentinel-1 data and processing

**Figure 9:**
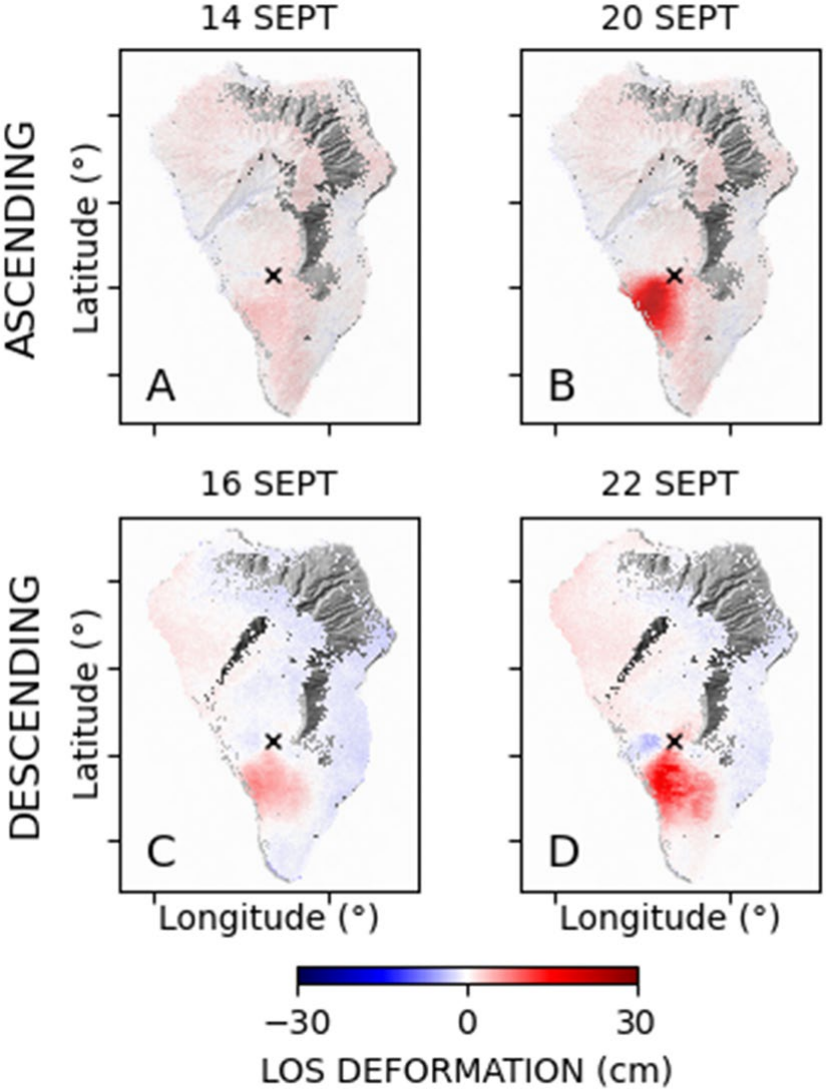
DInSAR deformation maps for La Palma in pre-and (**A** and **C** panel) and early-eruptive (**B** and **D** panel) phases. (**A** and **B**) represent the ascending orbit, while (**C** and **D**) represent the descending one. Black crosses show the location of the eruptive vents.

Two sets of synthetic aperture radar (SAR) images were acquired from complementary (ascending/descending) orbits between January and November 2021 through the constellation of twin radar sensors S-1A and S-1B, operating at the C band (wavelength of approximately 5.6 cm) and gathering images through the Interferometric Wide (I.W.) mode. They were independently processed using the multi-temporal interferometric SAR (Mt-InSAR) Small Baseline Subset (SBAS) technique^6^. The area covered by the used SAR images encompasses the whole island of La Palma (Fig. 9). The relevant parameters of the SAR datasets are listed in Table S1 (in supplementary material). For every detected coherent distributed scatterer (D.S.) on the ground, the corresponding time-series of the LOS-projected ground displacement components were generated. According to Berardino et al.^6^, Casu et al.^29^ and Lanari et al.^30^, the implemented SBAS processing chain operates on sequences of multi-look small baseline (S.B.) interferograms (in particular, 20 (range) × 4 (azimuth) looks were considered in our work) and includes specific steps for: (1) the space–time phase unwrapping^31^, (2) the estimation and compensation of phase artefacts in the generated SAR interferograms (i.e., the removal of residual topographic phases^6^), (3) the space–time noise-filtering of the sequence of small baseline multi-look SAR interferograms^25^ and (4) the compensation of the atmospheric phase screen (APS). Specifically, before their inversion, the noise-filtered, unwrapped interferograms were analysed to retrieve and compensate the APS components by implementing an ad-hoc strategy. First, on every single interferogram, the phase components that are spatially highly correlated with the topography were estimated and filtered out. Then, we applied the methodology proposed in Tymofyeyeva and Fialko^32^ that allows discriminating and filtering out the APS time uncorrelated components in a sequence of SAR images by implementing a stacking operation on couples of S.B. SAR interferograms made with a common SAR image and characterized by the same time span (i.e., temporal baseline). The estimated tropospheric and time-uncorrelated APS components were finally subtracted from the unwrapped interferograms inverted through the SBAS method to obtain the relevant ground displacement time series. The residual APS components were then further compensated with a space–time filter (e.g., see Ferretti et al.^33^, Berardino et al.^6^, Yang and Buckley^34^). Finally, the interferometric ground deformation products were geocoded, i.e., converted from radar to geographical coordinates. Figures S12 and S13 of the supplementary material show the generated LOS-projected mean displacement velocity maps from the ascending and descending orbit tracks, respectively. Then, we concentrated on the short interval between Sept. 8th and 28th, 2021, with an aim to analyse pre- and early-eruptive ground deformation. Accordingly, we extracted the layers corresponding to the selected SAR acquisitions from the generated LOS-projected ground displacement time series and performed the analyses detailed hereinafter.

Starting from the ground displacement time series obtained by separately processing through the SBAS approach the available ascending/descending S-1 SAR images, we focused on the retrieved cumulative ground deformations, calculated with respect to the first images of the two datasets acquired in January 2021. More specifically, the analyses addressed in our study refer to the time interval Sept. 8th–Sept. 28th, representing the core of the analyses shown in this study. Note that the obtained ground deformation values only represented the projection of the ground displacement along the relevant radar-to-target line-of-sight (LOS) directions and were calculated by assuming as a time reference the date of the first available SAR images of the ascending and descending time series, respectively, collected on the first days of January 2021. Figure 9 shows the pre- and early-eruptive cumulative LOS deformation maps for the processed ascending and descending orbits. The ground deformation and the magma ascent were rapid. In Fig. 9A, on Sept. 14th, the deformation shows a slight deformation. Two days later, on Sept. 16th, the descending orbit (Fig. 9C) captured a significant ground movement on the southern side of the forthcoming eruptive vent. On Sept. 19th at 14:02 GMT, the eruption started, but that day, Sentinel-1 did not acquire the data over the Canaries. One day after the eruption began, on Sept. 20th, the ascending orbit captured significant deformation in the southwestern side of the eruptive vent (Fig. 9B). The descending orbit that acquired the data on Sept. 22nd also captured considerable ground deformation (Fig. 9D). Its spatial deformation map differs slightly from the ascending orbit due to differences in the illumination geometries between the orbits and the different acquisition times that capture distinctive rapidly-evolving ground displacement signals from one date to another.

### Non-linear inversion for the shallow dike geometry

To determine the inclination of the shallow part of the dike, we performed a non-linear inversion using the analytical ground deformation model of Okada^15^, fixing the azimuth and letting the inclination, the width, the length and the opening to vary. We determined the best-fit model using the Nelder-Mead^35^ simplex algorithm.

### Geodetic imaging

We applied a non-linear inversion technique of the spatiotemporal pattern of the dike opening following the approach of D'Auria et al.^7^.

The dike opening function o(x,y,t) has been discretised into a set of 15 × 24 rectangular cells (Figs. 3, 4, 5 and 6) for each of the 8 DInSAR images used in this work. The computation of the Green's function for each cell has been performed within the finite element modelling environment COMSOL MultiPhysics®, using a 3D model that takes into account the topography and the bathymetry around the island. We used a lateral extent of the computational domain of 9 km. This width is sufficient to encompass all the areas affected by the eruptive phenomena. Furthermore, enlarging this length would negatively affect the resolution and the reliability of the final results. We performed the inversion using different sizes of the computational domain, obtaining similar results.

The opening function of the first image is constrained to 0, the first image being used as a reference for the rest of the dataset. Therefore, this allows the inverse problem formulation as a linear system for a total of 15 × 24 × 7 unknown. We used a second-order Tikhonov regularisation for both space and time. As with any inverse method, ours shows a trade-off between model resolution and fit with the data. The damping parameter controls this trade-off. Low damping values lead to lower misfit but unreliable noisy models. Conversely, high damping values lead to smoother models but high misfit values. In this work, we used the widely known L-curve approach^36^ to establish the optimal damping value. Since we used a positivity constraint for the opening function, we solved the inverse problem through a non-linear Sequential Least Squares Programming (SLSQP) algorithm.

The final models cannot justify all the observed ground deformation because of the intrinsic limitations related to the inverse method and the lack of details of the 3D model of the mechanical properties of the Cumbre Vieja volcano.

To check the resolution, we performed various checkerboard tests (see Figs. S3–S6 in the supplementary material) and a synthetic test with a realistic dike opening function (see Figs. S7–S10 in the supplementary material). We used the same data acquisition geometry as the actual data (GNSS three components, Ascending DInSAR and Descending DInSAR). The standard deviation of the Gaussian noise added to the synthetic data mimics those assumed for actual data: 10 mm for DInSAR, 5 mm for horizontal GNSS components, and 10 mm for vertical GNSS components.

### Supplementary Information

Supplementary Information.
