# Supplementary material for: Geodetic imaging of magma ascent through a bent and twisted dike during the Tajogaite eruption of 2021 (La Palma, Canary Islands)
Source: Sci Rep. 2024 Jan 2;14:212. doi: 10.1038/s41598-023-50982-9 (PMC10761690; doi:10.1038/s41598-023-50982-9)
Supplement: Supplementary file 1 — Supplementary Information. [file 41598_2023_50982_MOESM1_ESM.docx]

**Geodetic imaging of magma ascent through a bent and twisted dike during the Tajogaite eruption of 2021 (La Palma, Canary Islands)**

Monika Przeor^1,2^, Raffaele Castaldo^3^, Luca D’Auria^1,2^, Antonio Pepe^3^, Susi Pepe^3^, Takeshi Sagiya^4^, Giuseppe Solaro^3^, Pietro Tizzani^3^, José Barrancos Martínez^1,2^, Nemesio Pérez^1,2^

*^1^ Instituto Volcanológico de Canarias (INVOLCAN), Granadilla de Abona, Tenerife, Canary Islands, Spain*

*^2^ Instituto Tecnológico y de Energías Renovables (ITER), Granadilla de Abona, Tenerife, Canary Islands, Spain*

*^3^ Istituto per il Rilevamento Elettromagnetico dell’Ambiente (CNR-IREA), Napoli (ITALY)*

*^4^ Nagoya University, (JAPAN)*

**List of Figures:**

**Figure S1** shows the data, model, and residuals of deformation for the ascending orbit.

**Figure S2** shows the data, model, and residuals of deformation for the descending orbit.

**Figure S3** shows a checkerboard test for anomalies of 1 km.

**Figure S4** shows a checkerboard test for anomalies of 2 km.

**Figure S5** shows a checkerboard test for anomalies of 2.5 km.

**Figure S6** shows a checkerboard test for anomalies of 5 km.

**Figure S7** shows the synthetic tests for the true and retrieved model.

**Figure S8** shows the synthetic tests for the true and retrieved model.

**Figure S9** shows the synthetic tests for the true and retrieved model.

**Figure S10** shows the synthetic tests for the true and retrieved model.

**Figure S11** shows data and a synthetic model resulting from the Geodetic Imaging technique.

**Figure S12** shows the LOS-projected mean displacement velocity maps for the ascending orbit.

**Figure S13** shows the LOS-projected mean displacement velocity maps for the descending orbit.

**Figure S14** shows the horizontal cumulative displacement of GNSS station ARID.

**List of Tables:**

**Table S1** shows the SAR datasets' key parameters for data acquired in this study.


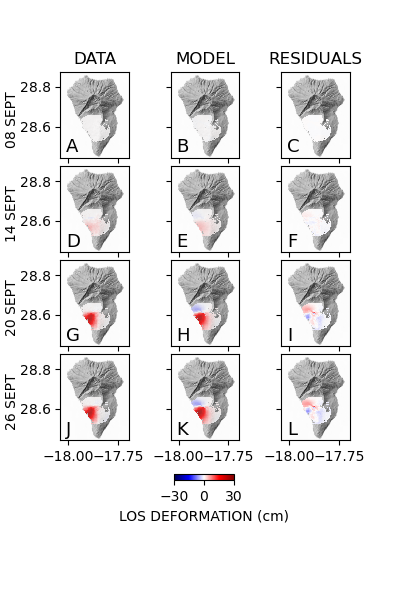


**Figure S1.** Data, model, and residuals for the source of the ground deformation on La Palma (columns 1, 2, and 3, respectively) for each data acquisition date of the ascending orbit.


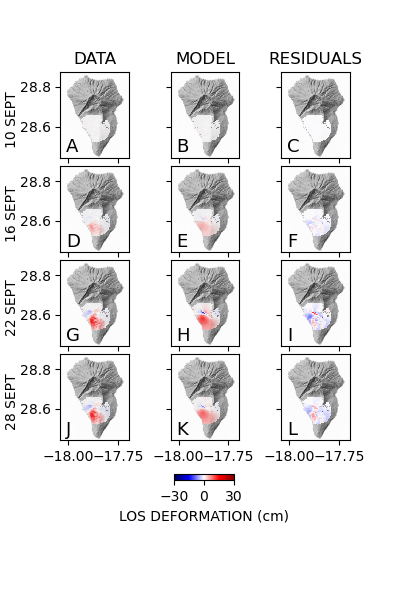
**Figure S2.** Data, model, and residuals for the source of the ground deformation on La Palma (columns 1, 2, and 3, respectively) for each of the data acquisition dates of the descending orbit


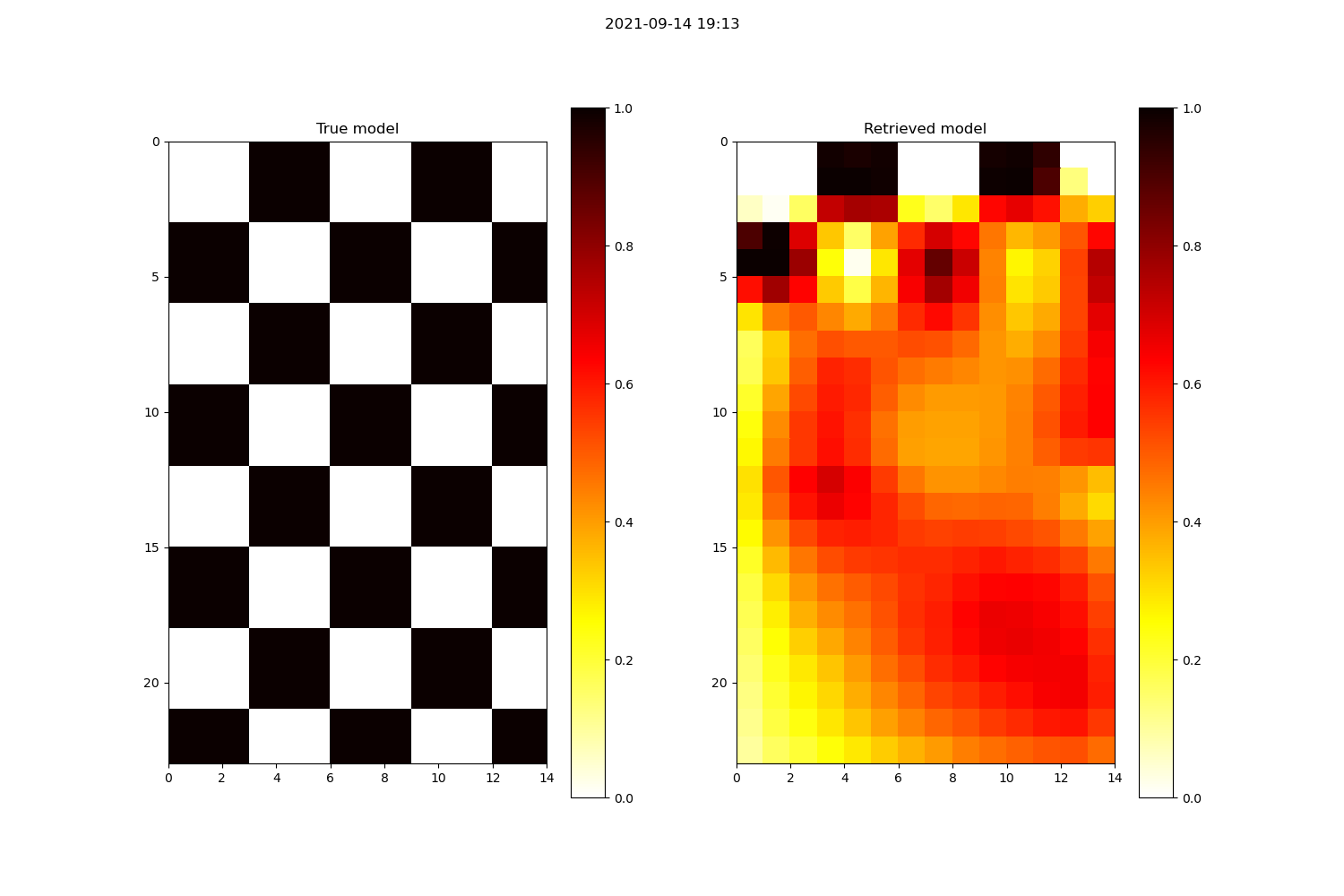


**Figure S3.** Checkerboard test for anomalies of 1 km. The left-hand side panel represents the true model while the right-hand side panel shows the retrieved model.
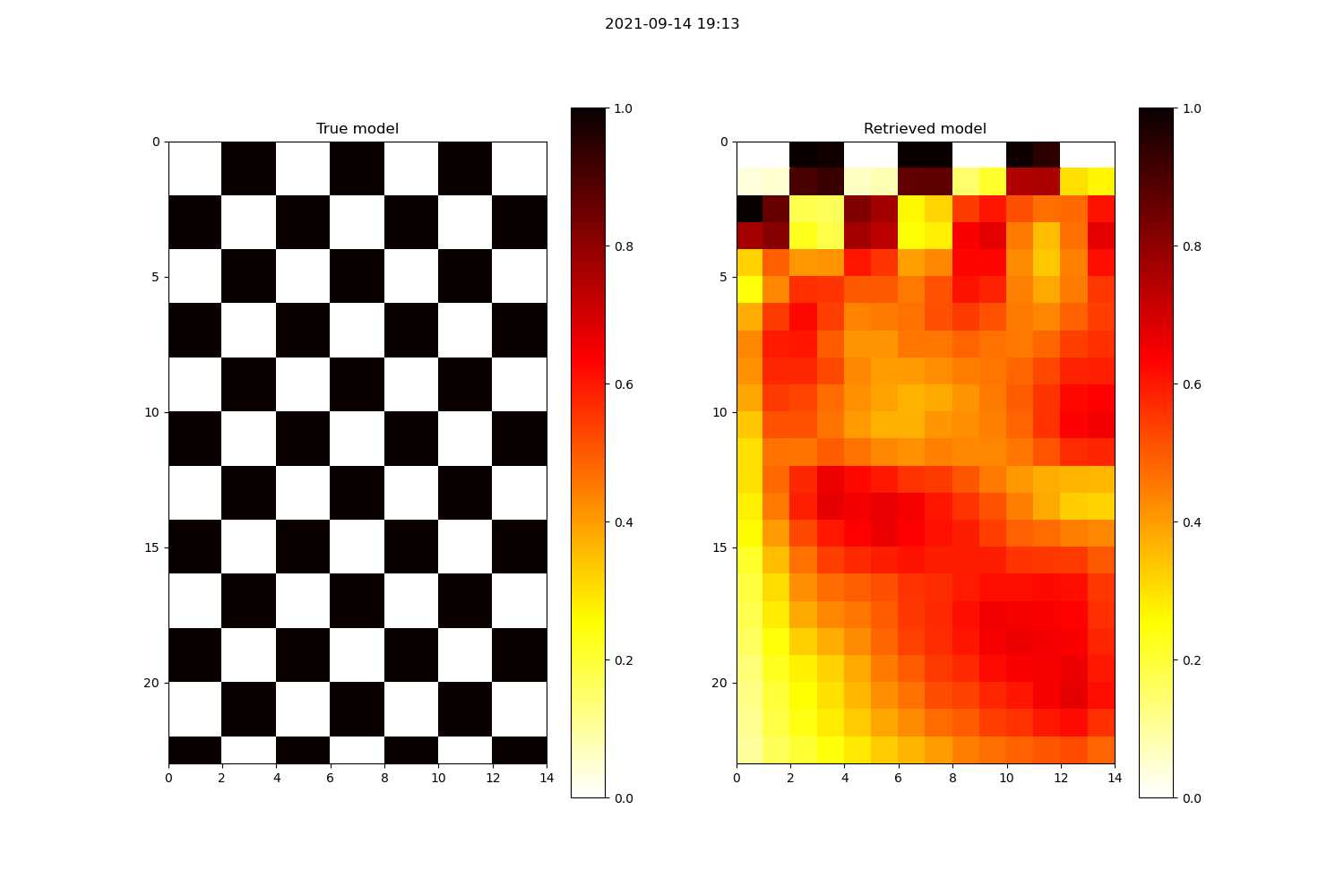


**Figure S4.** Checkerboard test for anomalies of 2 km. The left-hand side panel represents the true model while the right-hand side panel shows the retrieved model.


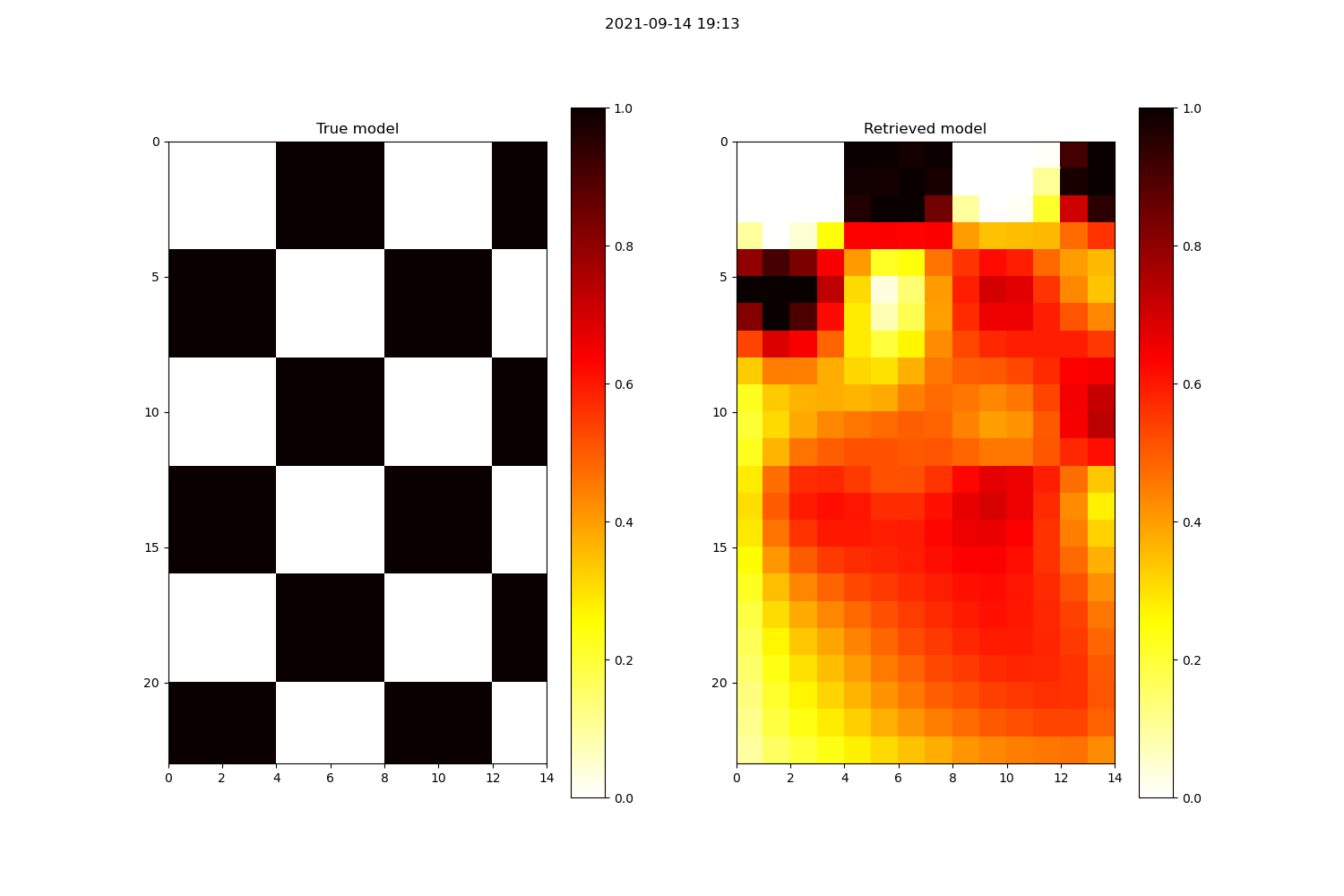


**Figure S5.** Checkerboard test for anomalies of 2.5 km. The left-hand side panel represents the true model while the right-hand side panel shows the retrieved model.


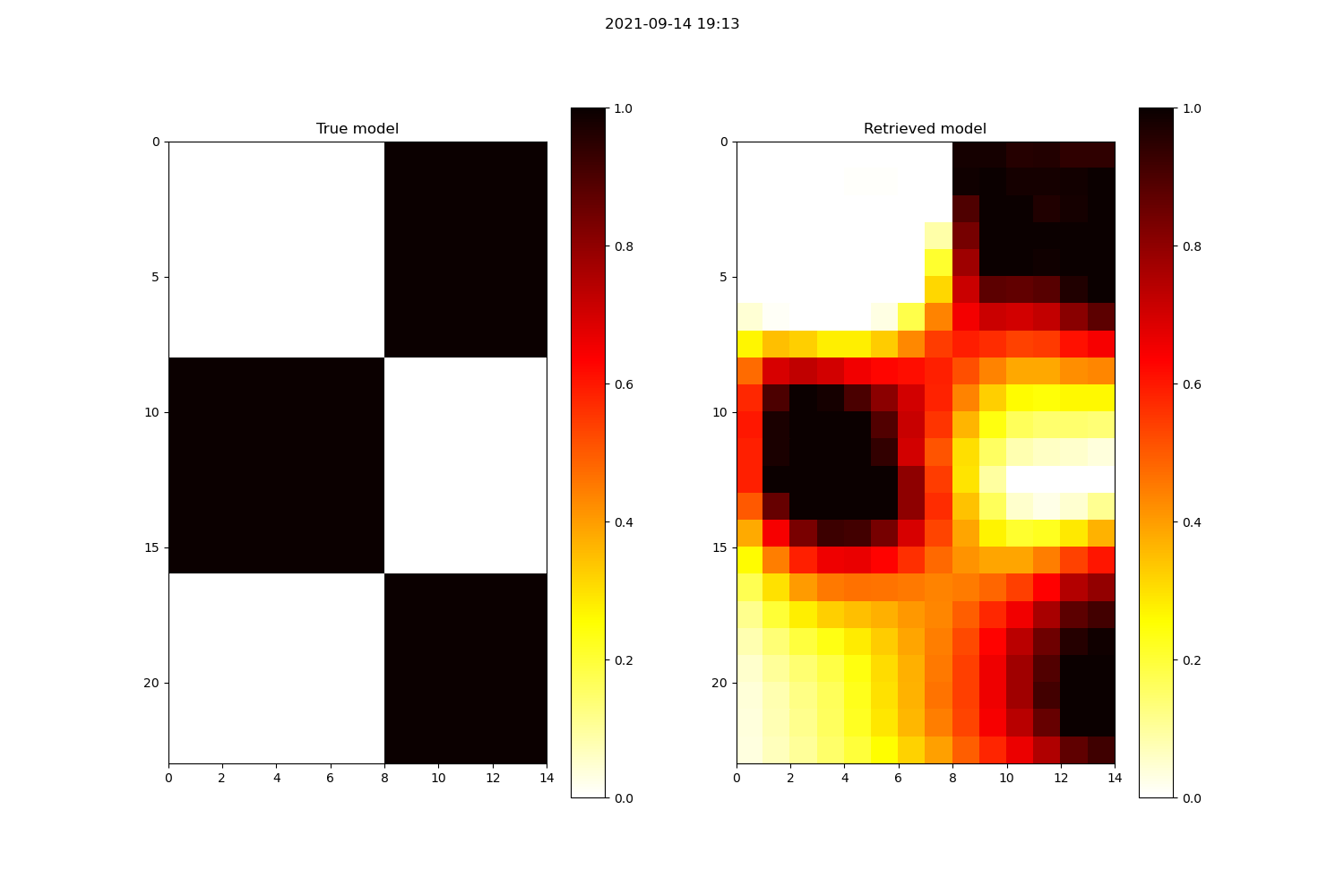


**Figure S6.** Checkerboard test for anomalies of 5 km. The left-hand side panel represents the true model while the right-hand side panel shows the retrieved model.


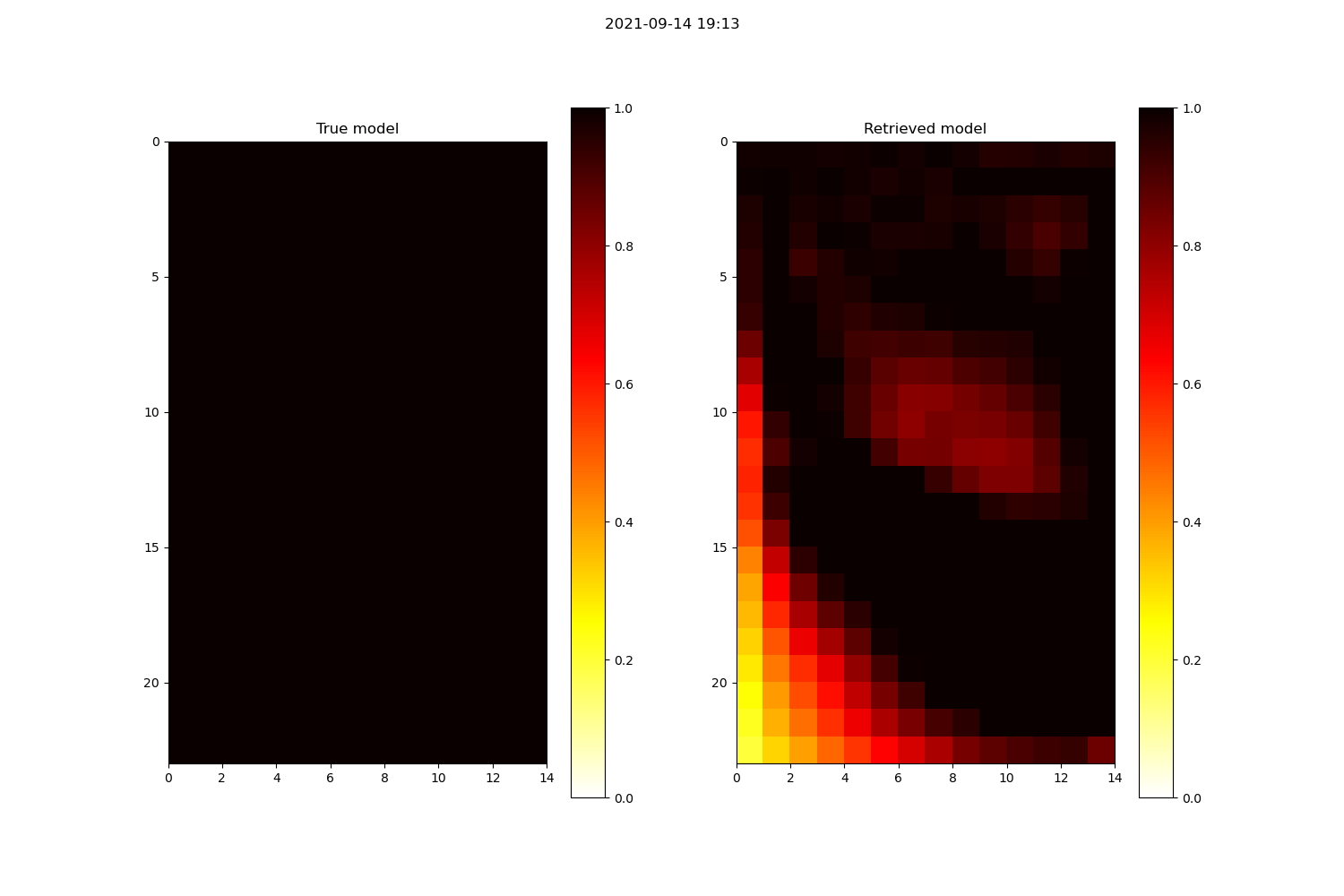


**Figure S7.** Synthetic tests for the true model (left-hand side panel) and the retrieved model (right-hand side panel).


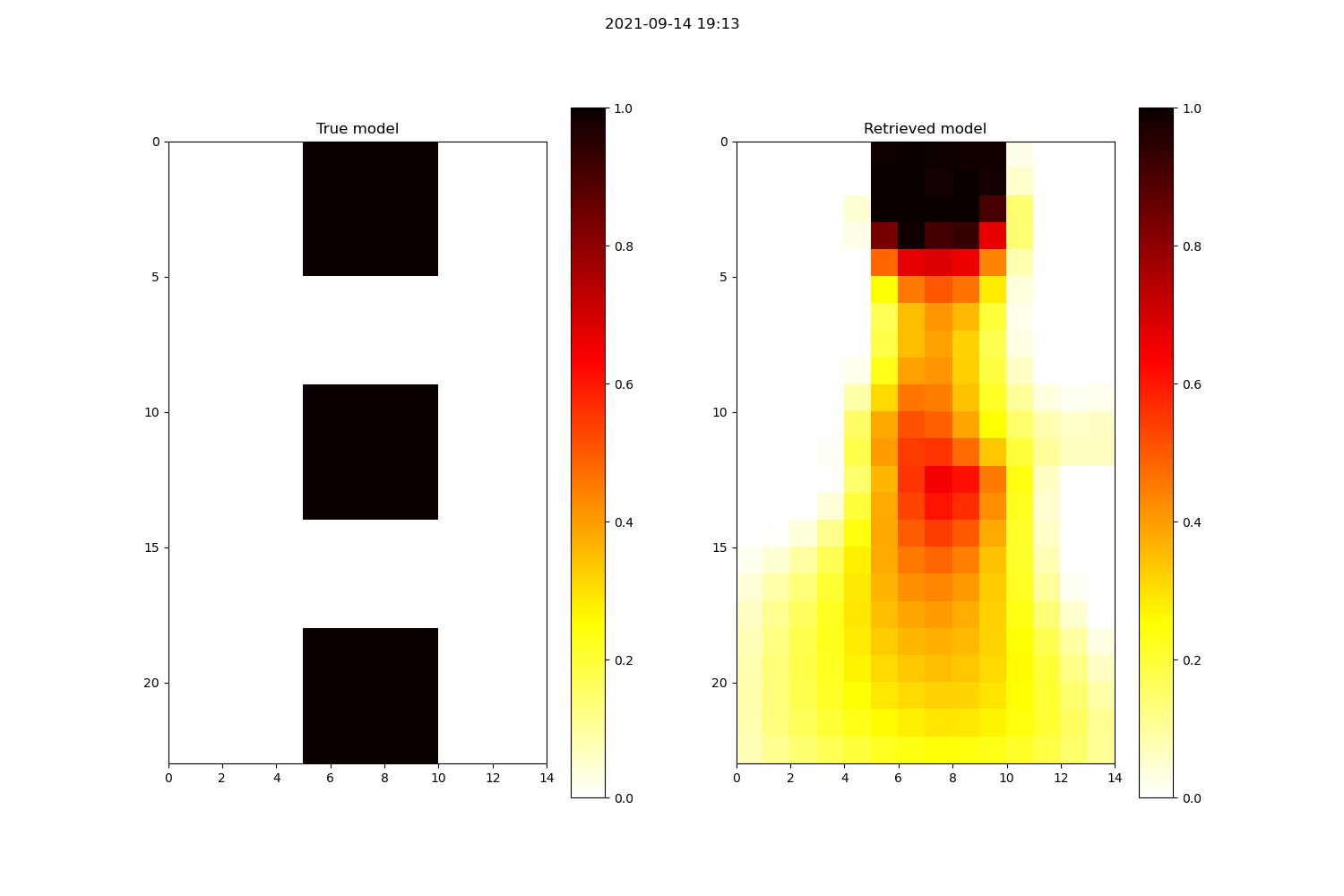


**Figure S8.** Synthetic tests for the true model (left-hand side panel) and the retrieved model (right-hand side panel).


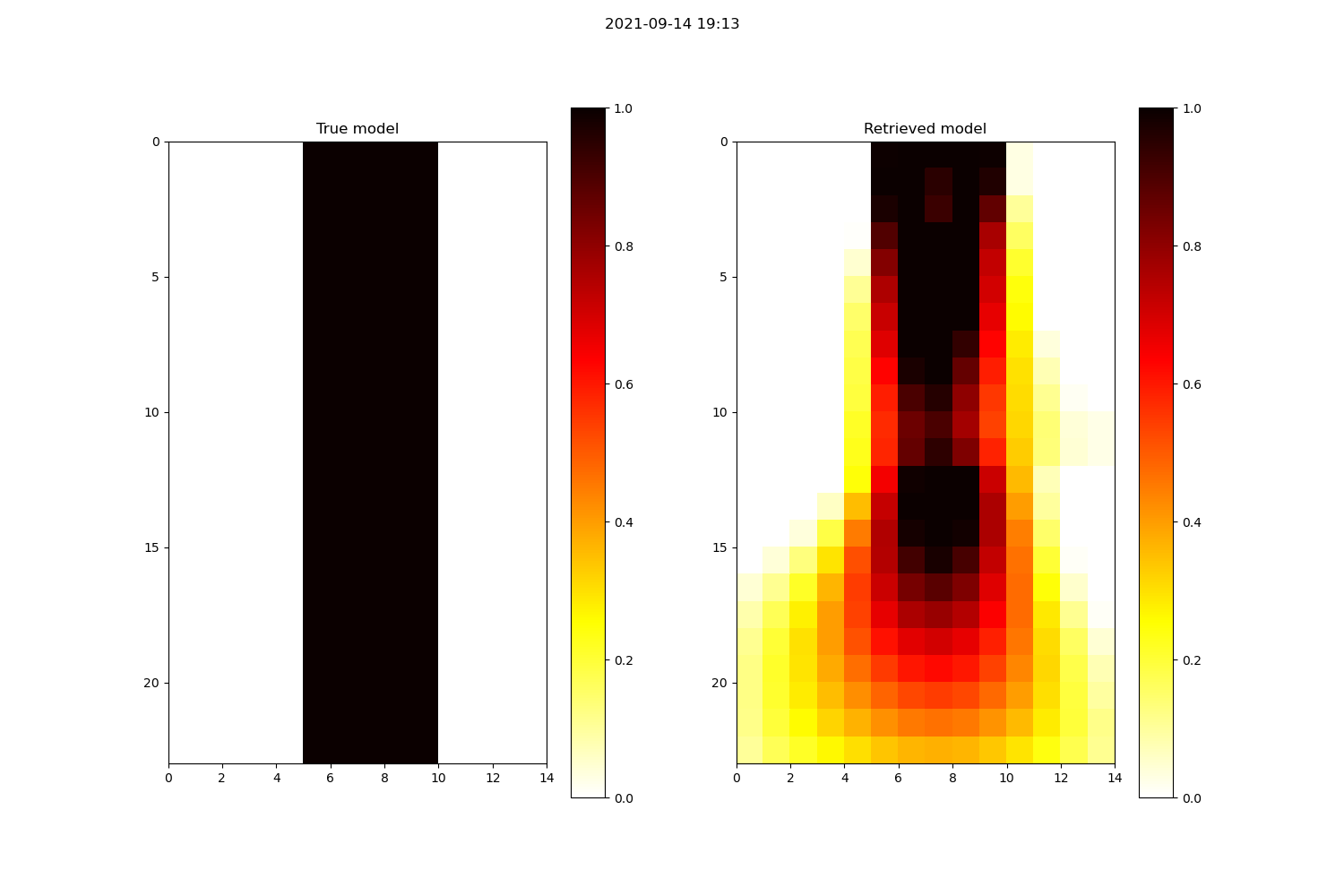


**Figure S9.** Synthetic tests for the true model (left-hand side panel) and the retrieved model (right-hand side panel).


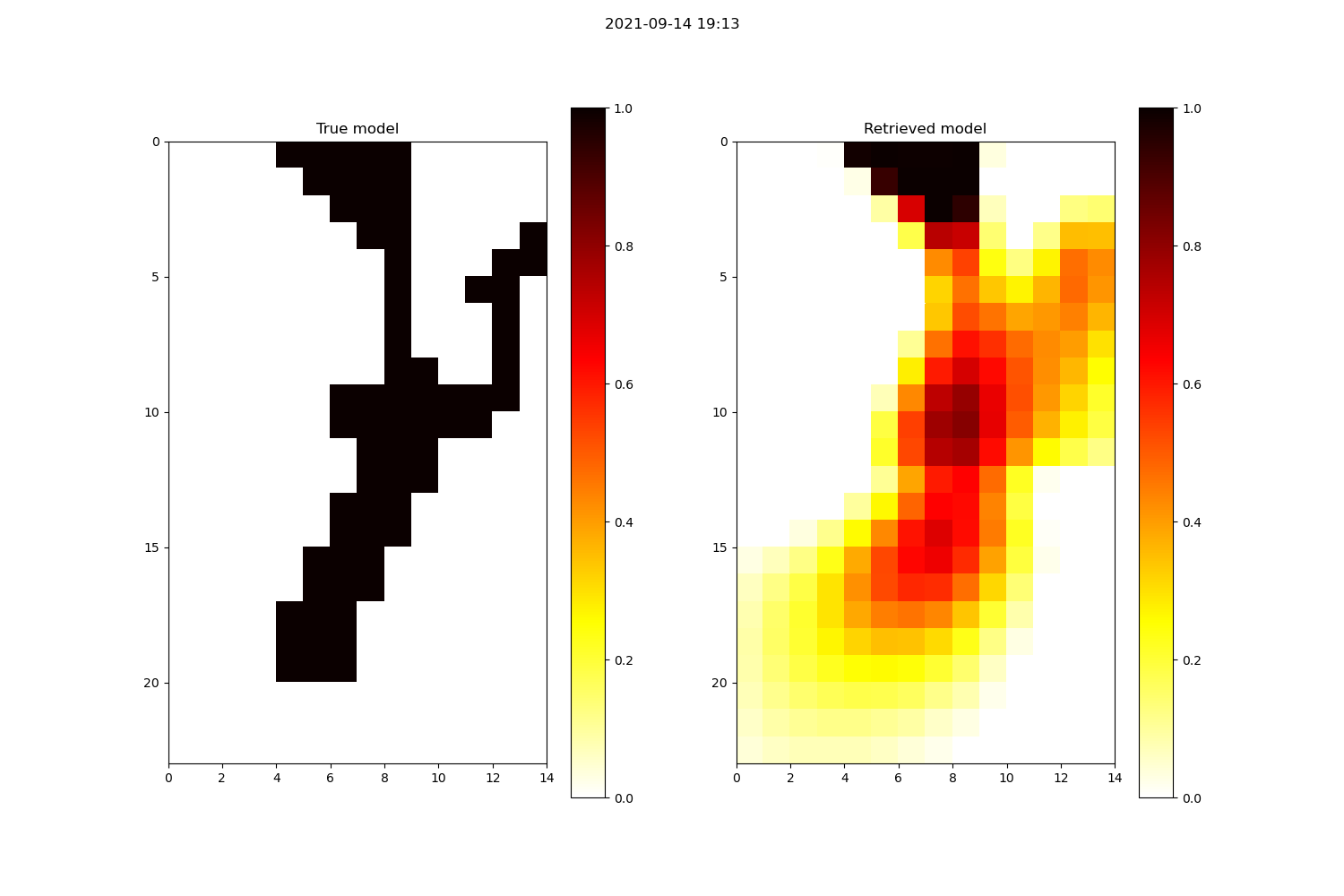


**Figure S10.** Synthetic tests for the true model (left-hand side panel) and the retrieved model (right-hand side panel).


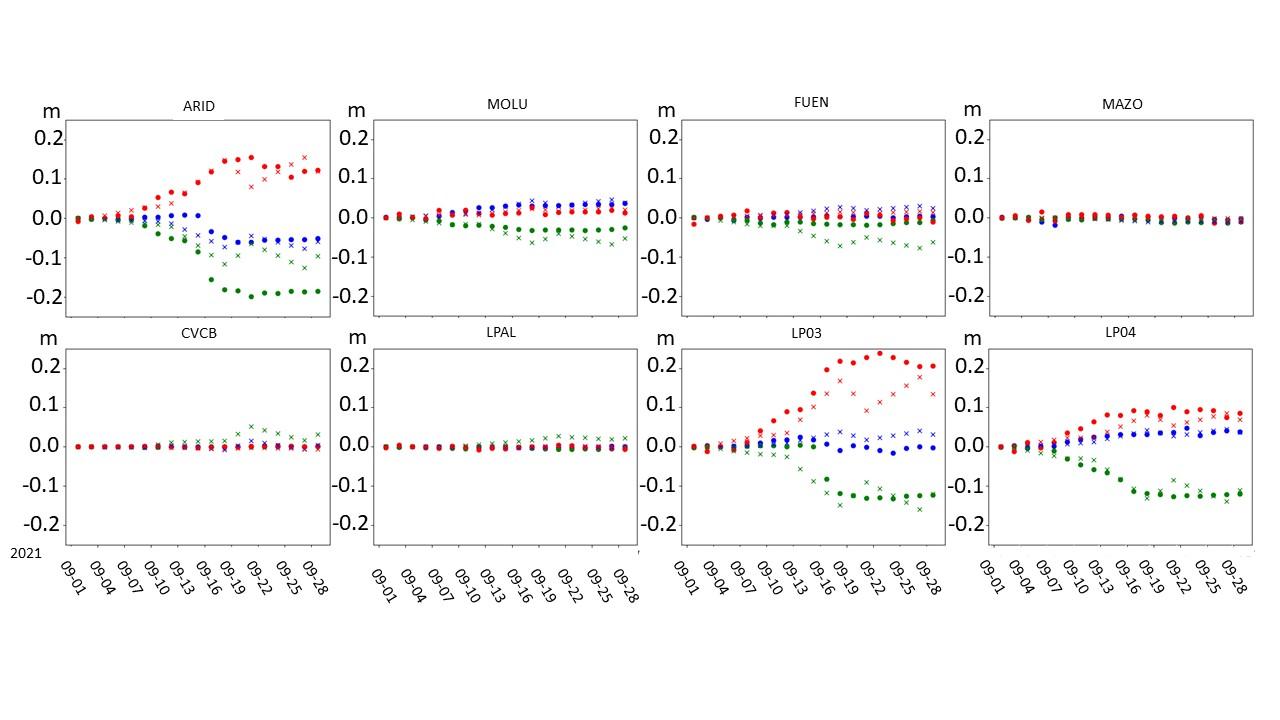


**Figure S11.** Data and synthetic model resulting from the Geodetic Imaging technique, for the three components of the GNSS data for all the stations used for this study. Each point represents the daily data solution while each x-symbol represents the synthetic model solution. Red, blue, and green colors represent the vertical, N-S, and E-W components, respectively.


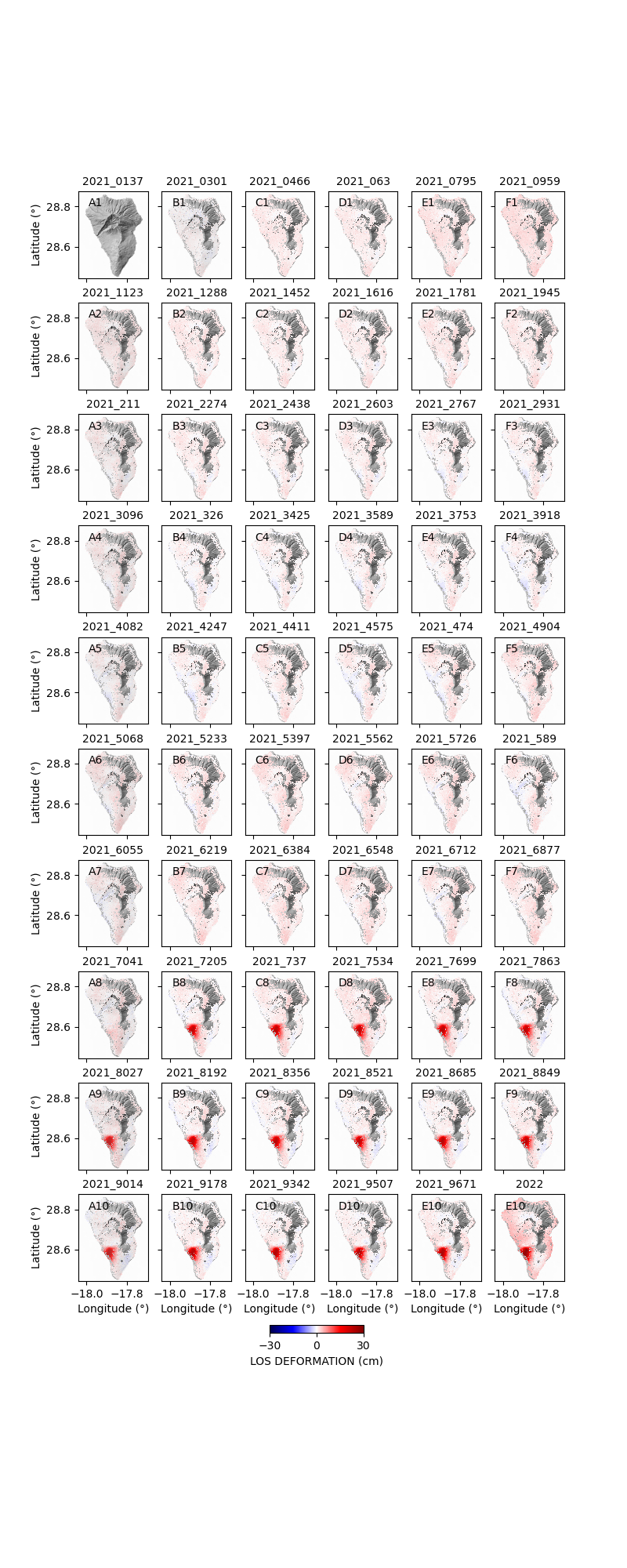


**Figure S12.** LOS-projected mean displacement velocity maps for the ascending orbit tracks from January to November 2021.


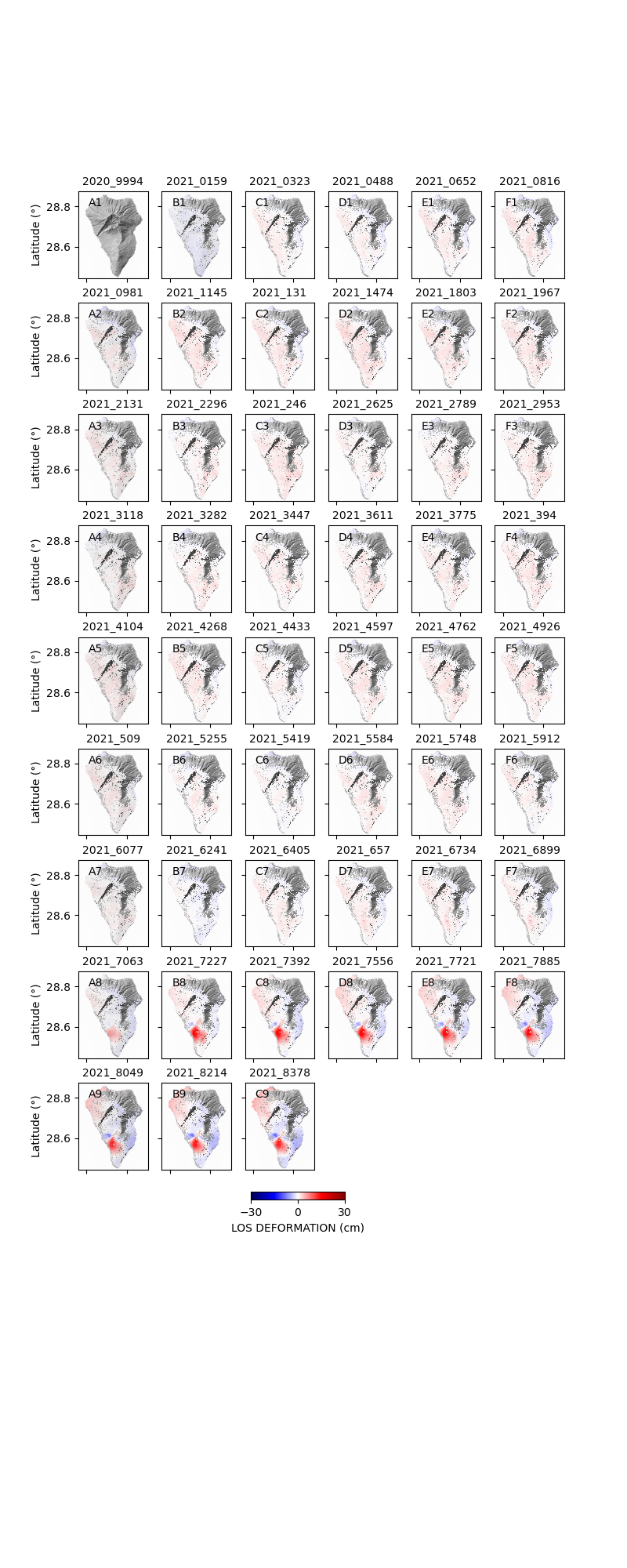


**Figure S13.** LOS-projected mean displacement velocity maps for the descending orbit tracks from January to November 2021.


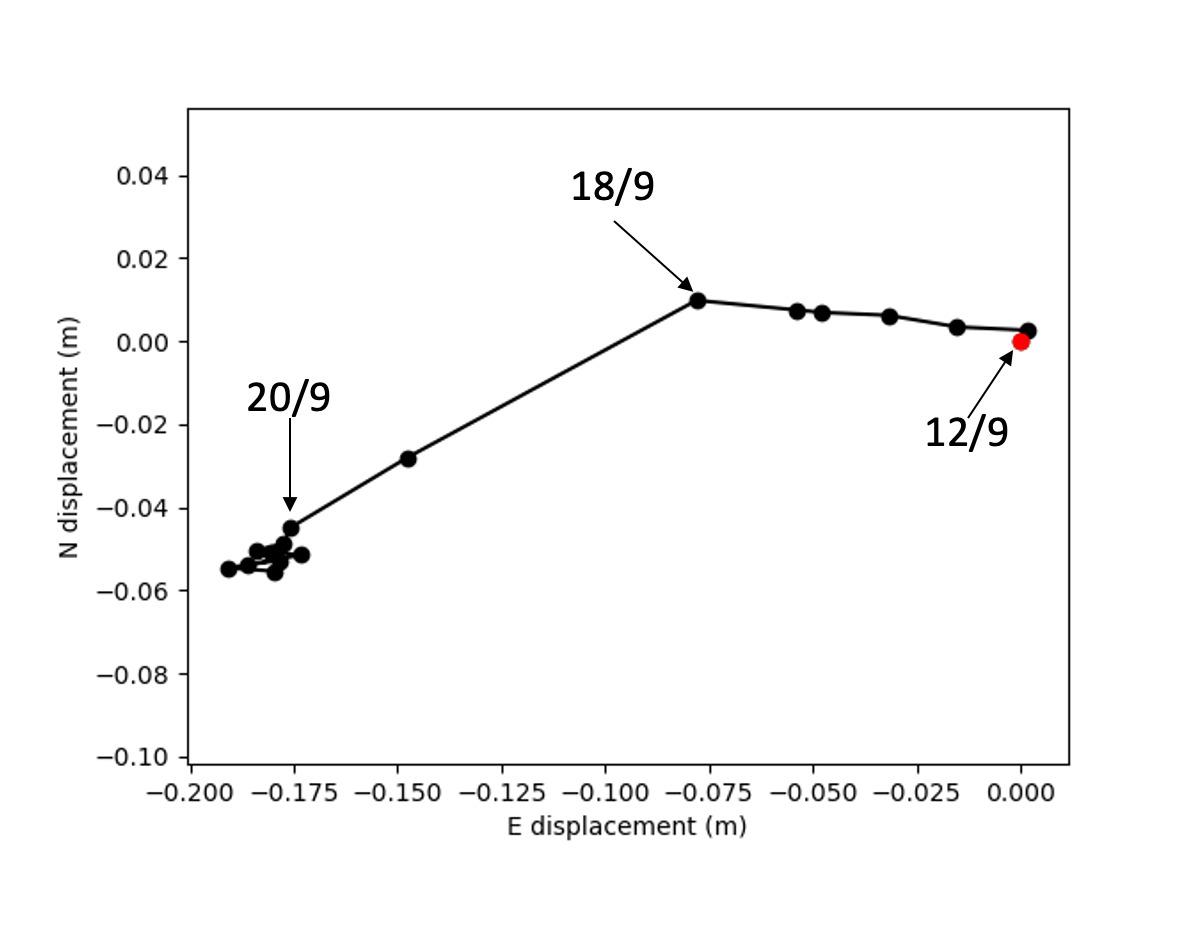


**Figure S14.** Horizontal cumulative displacement of GNSS station ARID. We represent the cumulative horizontal displacement from the 12th to the 28th of September 2021. Each point represents a daily solution. The red point marks the beginning of the time series. We annotated relevant dates as discussed in the text.

| Main Sentinel-1 SAR data parameters | | |
| --- | --- | --- |
| Satellite | Sentinel-1A | Sentinel-1B |
| Orbit | Ascending | Descending |
| Beam mode | IW | |
| Path | 60 | 169 |
| Frame | 87 | 498 |
| Number of SLC images | 51 | 51 |
| Time interval | January 2021-November 2021 | January 2021-November 2021 |

Table S1. SAR datasets' key parameters for data acquired in this study.
